# Phycobilins Versatile Pigments with Wide-Ranging Applications: Exploring Their Uses, Biological Activities, Extraction Methods and Future Perspectives

**DOI:** 10.3390/md23050201

**Published:** 2025-05-04

**Authors:** Celestino García-Gómez, Diana E. Aguirre-Cavazos, Abelardo Chávez-Montes, Juan M. Ballesteros-Torres, Alonso A. Orozco-Flores, Raúl Reyna-Martínez, Ángel D. Torres-Hernández, Georgia M. González-Meza, Sandra L. Castillo-Hernández, Marcela A. Gloria-Garza, Miroslava Kačániová, Maciej Ireneusz-Kluz, Joel H. Elizondo-Luevano

**Affiliations:** 1 Facultad de Agronomía, Universidad Autónoma de Nuevo León, Francisco I. Madero S/N, Ex Hacienda el Canada, Escobedo 66050, Nuevo León, Mexico; celestino.garciagm@uanl.edu.mx; 2 Facultad de Ciencias Biológicas, Universidad Autónoma de Nuevo León, Av. Universidad S/N, Cd. Universitaria, San Nicolás de los Garza 66455, Nuevo León, Mexico; diana.aguirrecvz@uanl.edu.mx (D.E.A.-C.); abelardo.chavezmn@uanl.edu.mx (A.C.-M.); juan.ballesterostr@uanl.edu.mx (J.M.B.-T.); aorozcof@uanl.edu.mx (A.A.O.-F.); angel.torreshr@uanl.edu.mx (Á.D.T.-H.); georgina.gonzalezm@uanl.mx (G.M.G.-M.); sandra.castilloh@uanl.mx (S.L.C.-H.); 3 Facultad de Ciencias Químicas, Universidad Autónoma de Nuevo León, Av. Universidad S/N, Cd. Universitaria, San Nicolás de los Garza 66455, Nuevo León, Mexico; gustavo.reynamr@uanl.edu.mx; 4 Facultad de Odontología, Universidad Autónoma de Nuevo León, Dr. Eduardo Aguirre Pequeño S/N, Monterrey 64460, Nuevo León, Mexico; marcela.gloriagz@uanl.edu.mx; 5 Institute of Horticulture, Faculty of Horticulture and Landscape Engineering, Slovak University of Agriculture, Tr. A. Hlinku 2, 94976 Nitra, Slovakia; 6 School of Medical & Health Sciences, University of Economics and Human Sciences in Warsaw, Okopowa 59, 01-043 Warszawa, Poland; 7 Andrzej Frycz Modrzewski Krakow University, Gustawa Herlinga-Grudzińskiego 1, 30-705 Kraków, Poland

**Keywords:** cyanobacteria, pigments, phycobilins, phycocyanin, allophycocyanin, phycoerythrin, phycobiliviolin

## Abstract

Phycobiliproteins (PBPs), captivating water-soluble proteins found in cyanobacteria, red algae, and cryptophytes, continue to fascinate researchers and industries due to their unique properties and multifaceted applications. These proteins consist of chromophores called phycobilins (PBs), covalently linked to specific protein subunits. Major phycobiliproteins include phycocyanin (PC), allophycocyanin (APC), and phycoerythrin (PE), each distinguished by distinct absorption and emission spectra. Beyond their colorful properties, PBs exhibit a broad spectrum of biological activities, including antibacterial, antifungal, antiviral, and antidiabetic effects, making them valuable for pharmaceutical, biotechnological, and medical purposes. The extraction and purification methods for PBs have been optimized to enhance their bioavailability and stability, opening new avenues for industrial production. For this review, a comprehensive literature search was conducted using scientific databases such as PubMed, Scopus, and Web of Science, prioritizing peer-reviewed articles published between 2000 and 2025, with an emphasis on recent advances from the last five years, using keywords such as “phycobiliproteins”, “phycobilins”, “bioactivities”, “therapeutic applications”, and “industrial use”. Studies were selected based on their relevance to the biological, technological, and pharmacological applications of PBPs and PBs. This review explores the diverse applications of PBs in therapeutic, diagnostic, and environmental fields, highlighting their potential as natural alternatives in the treatment of various diseases. The future perspectives for PBs focus on their incorporation into innovative drug delivery systems, biocompatible materials, and functional foods, presenting exciting opportunities for advancing human health and well-being.

## 1. Introduction

Phycobilins (PBs) are unique and vibrant pigments in various photosynthetic organisms, particularly in cyanobacteria and red algae [1,2]. These pigments are crucial in capturing and transferring light energy for photosynthesis [3]. However, beyond their photoreceptive functions, PBs have garnered significant attention due to their diverse biological activities and potential applications in various fields.

Certain groups of algae, particularly cyanobacteria, red algae, glaucophytes, and some cryptophytes, possess supramolecular pigment complexes known as phycobilisomes (PBSs), which function as antennas on the external membrane of thylakoids. These are composed of aggregated phycobiliprotein (PBP) molecules that act as accessory systems for light harvesting. These so-called phycobilins are unique and vibrant pigments crucial for capturing and transferring light energy for photosynthesis [4].

To date, more than 10 distinct PBs have been identified, each composed of different α and β polypeptide subunits [5]. These proteins are classified based on their wavelength and the presence of various types of chromophores. The types of PBP include phycoerythrins (PEs), phycoerythrocyanins (PECs), phycocyanins (PCs), and allophycocyanins (APCs) [5,6,7].

**Figure 1:**
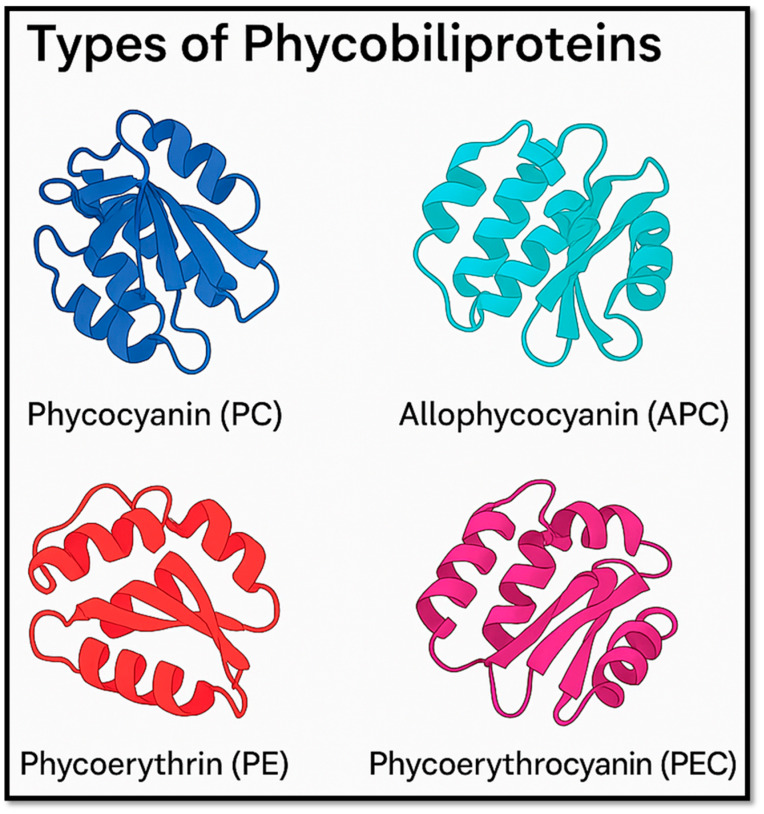
Types of phycobiliproteins (PBPs).

The biosynthesis of PBs is derived from heme metabolism, starting with the production of biliverdin by heme oxygenase (HO). Biliverdin can then be reduced by the family of phycobilin reductases (PcyA, PebA, PebB, and PebS), which further reduce it into other types of PBs. This process confers on them a unique chemical structure that enables them to exhibit fascinating biological properties [8]. Moreover, since the exogenous gene of ferredoxin oxidoreductase is associated with phycocyanin and can be expressed in some bacteria, phycobiliproteins can be produced on a large scale through biosynthesis in *Escherichia coli* [7]. Beyond their photoreceptive functions, PBs have attracted significant attention due to their diverse biological activities and potential applications in various fields, ranging from biotechnology to medicine. This review explores the types (Figure 1), uses, biological activities, examples, extraction methods, and future perspectives of PBs.

## 2. Selection Methods

To prepare this review, a systematic search of updated scientific literature published between 2000 and 2025 was conducted. The review focuses on the classification of phycobiliproteins (PBPs) and their chromophores (phycobilins, PBs), as well as their biological activities, extraction and purification methods, pharmaceutical and biotechnological applications, and industrial perspectives. The information was gathered from well-established databases, including PubMed, ScienceDirect, Scopus, SpringerLink, Web of Science, and Google Scholar. To refine the search and ensure the inclusion of the most relevant studies, Boolean operators such as AND, OR, and NOT were used. For example:

1. “phycobiliproteins” OR “phycobilins” to retrieve results that included either term, broadening the search scope.
2. “phycobiliproteins OR phycobilins” AND “biological activity” to find articles discussing the bioactivity of these compounds.
3. “phycobilins” AND (“extraction methods” OR “purification techniques”) to locate information on technological approaches.
4. “phycobiliproteins” NOT “chlorophyll” to exclude unrelated pigment studies.

Only peer-reviewed articles, reviews, and book chapters published in English or Spanish and within the specified period (2000–2025) were included. Studies were selected based on their contribution to the understanding of the structure, functions, and practical applications of PBPs and PBs. This systematic strategy ensured a comprehensive, up-to-date, and thematically relevant overview of the current knowledge on these natural pigments.

## 3. Types of Phycobiliproteins

### 3.1. Phycoerythrin

**Figure 2:**
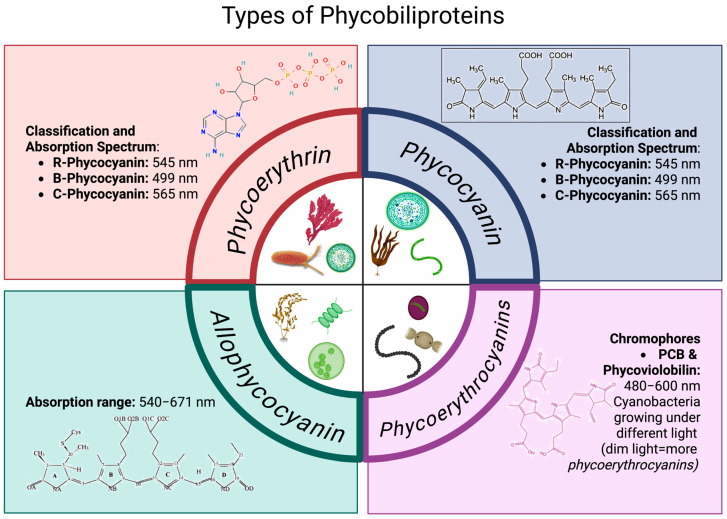
Types of phycobiliproteins (PBPs) and absorption range in nm.

Phycobiliproteins (PBPs), in the form of Phycoerythrin (PE), are the most abundant in red algae and some cyanobacteria [2,9]. These proteins contain chromophores that present a coloration ranging from red to fluorescent purple and are commonly extracted from microalgae cultures such as *Rhodella* spp., *Bangia* spp., and *Porphyridium* spp. [6]. Their absorbance spectrum exhibits a peak between 490 and 671 nm, and based on their spectral characteristics and taxonomic origin, they are classified into three major groups (Figure 2):

1. R-Phycocyanin (R-PE), with absorbance peaks at 499 and 565 nm and a shoulder at 545 nm.
2. B-Phycocyanin (B-PE), with absorbance peaks at 545 and 565 nm and a shoulder at 499 nm.
3. C-Phycocyanin (C-PE), with an absorbance peak at 565 nm.

The prefixes “R”, “B”, and “C” are assigned based on the taxonomic origin of the species from which they are derived: R-PE is obtained from rhodophytes, B-PE from Bangiales [5], and C-PE is most commonly found in cyanobacteria [2,9]. R-PE is a disc-shaped complex consisting of three monomers, each composed of two subunits bound by electrostatic interactions (αβ). In the middle of this complex, there are trimers (αβ)_3_, or two trimers that combine to create greater stability. These proteins may also contain a γ subunit, which often results in the formation of hexametric structures [5,9,10].

Within C-PE, two types have been identified: C-PE-I and C-PE-II, which differ primarily in the number and type of chromophores they contain. C-PE-I consists of five PEB molecules attached to residues at positions a-84, a-140 or 143, b-84 or 82, b-50 or b-61, and b-155 or 159. In contrast, C-PE-II has the same arrangement of PEB molecules at the corresponding positions but also contains a PUB molecule attached to cysteine at position a-75 [4].

### 3.2. Phycoerythrocyanins

Certain species of cyanobacteria exhibit a fourth group of biliproteins known as phycourobilin-cyanins (ficeoeritrocianins), whose chromophores are PCB and phycoviolobilin [5,11]. These proteins present an absorbance range from 480 to 600 nm. Functionally, they serve as an alternative to phycoerythrin [12], although structurally, they resemble phycocyanins due to the nature of their chromophore. This chromophore imparts an orange color [5], while other studies mention that their hues range from purple to intense blue [10,13]. Phycourobilin-cyanin typically occurs in trimeric (αβ)_3_ or hexameric (αβ)_6_ forms, and its chromophore is phycoviolobilin [10]. One of the key features of phycourobilin-cyanin is that its abundance is closely related to the intensity and type of light to which the cyanobacteria were exposed during growth. Its concentration significantly increases under conditions of dim light, exhibiting a green color [4,10].

### 3.3. Phycocyanin

Phycocyanin (PC) and PE are the most abundant phycobiliproteins in nature and occur as a complex mixture of monomers, trimers, hexamers, and other oligomers. Since their main chromophore is phycocyanobilin, they exhibit blue hues and may contain phycoerythrin. The molecular mass of these proteins ranges from 44 to 260 kDa, and they have an absorption range from 569 to 625 nm [10,11]. There are two main types of phycocyanins (PCs):

C-Phycocyanin (C-PC): The chromophore responsible for the characteristic blue color of this protein is phycocyanobilin (PCB) (C_33_H_38_N_4_O_6_), which has an absorption maximum between 615 and 620 nm. Phycocyanobilin is a linear tetrapyrrolic prosthetic group covalently bound to specific cysteine residues via thioether bonds in the protein’s polypeptide chain. In C-phycocyanin, three phycocyanobilin chromophores are found: one is attached to cysteine 84 of the α chain, and the other two are attached to cysteines 84 and 155 of the β chain [11,14].

R-Phycocyanin (R-PC): This type of phycocyanin is unique because it contains both phycocyanobilin and phycoerythrobilin. Cyanobacteria are a major source of this protein, where it can account for up to 20% of their dry weight. R-phycocyanin is water-soluble and can be extracted as a protein–pigment complex. It is primarily obtained from certain cyanobacteria and red algae [14].

According to Dagnino-Leone et al. (2022) [4], R-PCs exhibit different chromophores depending on the origin of the PBP:

1. R-PC-I: These are the most abundant and are found in red algae. They were the first phycocyanins to be spectroscopically characterized. This protein contains three chromophores of phycocyanobilin (PCB) in its structure: one attached to cysteine 84 of the α chain, and the other two to cysteine 84 and 155 of the β chain. The absorption spectrum of this R-PC shows two peaks of maximum absorption: the first, at less than 555 nm, is attributed to the PEB molecule, and the second, higher peak, associated with PCB molecules, is at 646 nm.
2. R-PC-II: This was the first phycocyanin from cyanobacteria in which a PEB molecule was reported. This type of phycocyanin has a PEB molecule attached to cysteine 84 of the α subunit, while the β subunit contains a PCB molecule attached to cysteine 84 and a PEB molecule at cysteine 155. Its absorption spectrum shows three peaks at 533, 545, and 615 nm. Its fluorescence emission is at 646 nm.
3. R-PC-III: This phycocyanin contains two molecules of PCB and one of PEB. Unlike the previous R-PCs, the PCB molecules are attached at residues 84 and 153 of the β subunit, while the PEB molecule is attached at residue 84 of the α subunit. Its absorption spectrum shows two peaks of maximum absorption, one at 560 nm and another with maximum intensity at 603 nm. The maximum fluorescence emission of this phycocyanin is at 648 nm.
4. R-PC-IV: This phycocyanin differs from the other three types by having a PUB molecule attached at the 84 positions of the α subunit and two PCB molecules at residues 84 and 155 of the β subunit. Its absorption spectrum shows two maximum peaks, one at 490 nm and another at 592 nm. The maximum emission of this protein is at 644 nm.
5. R-PC-V: This protein is characterized by having three different types of PUB chromophores at the 84 positions of the α subunit, one PCB molecule at residue 82, and one PEB molecule at residue 153 of the β subunit. Its absorption spectrum shows three maximum peaks: one at 495 nm, one at 540 nm, and one at 590 nm. The fluorescence emission is at 640 nm.

### 3.4. Allophycocyanin

Allophycocyanin (APC) are characterized by their blue color with a greenish hue, and their chromophore is phycocyanobilin (PCB) [11,14]. The biological unit of this protein is trimeric, consisting of α and β subunits, forming a complex (αβ)_3_, which spectroscopically makes it the simplest phycobiliprotein. They have a maximum absorption range of 540–671 nm [4,10].

### 3.5. Habitat Influence on Phycobiliprotein (PBPs) Production

The diversity of marine habitats inhabited by cyanobacteria significantly influences the production and characteristics of phycobilins (PBs), key pigments for their adaptation to different light conditions [15,16]. In open waters, light availability and depth modulate the composition of PBs, with species such as *Synechococcus* and *Prochlorococcus* optimizing light capture in oligotrophic environments [17]. In contrast, benthic cyanobacteria, such as those forming stromatolites and coastal biofilms, adjust their phycobilin production to light variability and interactions with other organisms. These ecological adaptations are crucial for understanding the variability in the quality and quantity of PBs, which in turn impacts their biotechnological applications [16,18].

The exploration of phycobilins for various biotechnological applications requires a deep understanding of the marine cyanobacteria’s living environment [15]. Abiotic factors such as light, temperature, and salinity vary drastically among marine habitats, influencing phycobilin biosynthesis and stability [17]. For example, deep-sea cyanobacteria may produce phycobilins with unique spectral properties, potentially useful in biomedical and photonic applications [17]. Understanding how climate change and pollution affect these habitats is essential to ensuring the sustainable supply of high-quality phycobilins for future applications.

## 4. Industrial Applications

### 4.1. Biotechnology

Phycobiliproteins (PBPs) possess inherent autofluorescence, making them valuable in diverse applications such as molecular probes, immunobiology, confocal microscopy, fluorescent spectroscopy, flow cytometry, and fluorescence microscopy for diagnostics and biomedical research [4]. The fluorescence spectrum exhibits multiple emission wavelengths, including PC (red spectrum, 630–650 nm), PE (yellow spectrum, 568–575 nm), PEC (orange, 600–610 nm), R-PC (red, 635–638 nm), R-PE (yellow spectrum, 570–575 nm), and APC (light red, 660–665 nm) [4]. PBPs exhibit distinct spectral properties, such as high excitation and emission spectra, low interference, water solubility, and high quantum yield, establishing them as a novel class of molecular tags in fluorescent applications compared to synthetic fluorophores. As a result, there is a growing demand for natural fluorescent proteins in the market, especially in biotechnology, pharmaceuticals, biomedical, and experimental molecular biology. When conjugated to molecules with biological specificity (e.g., immunoglobulins, avidin, and biotin), PBPs serve as excellent reagents for two-color fluorescence analysis of single cells using a fluorescence-activated cell sorter (FACS). Phycoerythrin (PE) stands out as the most widely used fluorescent probe and forms the basis of the detection system in Affymetrix chips (DNA microarrays). PBPs bound to monoclonal and polyclonal antibodies make them valuable fluorescent markers for cell sorting and the investigation of surface cell antigens. PE can also be combined with Cy5/Cy7 (Indodicarbocyanine/Indotricarbocyanine) dye for easy identification and visualization in molecular biology. Additionally, it can be integrated with streptavidin proteins for the application of biotin-integrated DNA probes. Despite these applications, it is noted that in the visible range of the spectrum, detecting biomolecules in living body tissue is not feasible, limiting its effectiveness for certain target applications. Several companies, including Boehringer Ingelheim (Ingelheim, Germany), Sigma-Aldrich^®^ (Merck KGaA, Darmstadt, Germany), and Thermo Fisher Scientific (Waltham, MA, USA), are actively producing and marketing PC/PE/APC molecular probes for various life sciences applications [15,19,20].

### 4.2. Food and Beverage Industry

The food and beverage industry is actively searching for natural alternatives to synthetic dyes, given the typical toxic, carcinogenic, or unsafe nature of synthetic dyes [21]. This demand is driven by shifts in consumer preferences towards natural, minimally processed foods that offer health benefits through bioactive properties [22]. However, incorporating natural dyes into foods faces challenges due to the composition of the food matrix, including interactions with proteins, polysaccharides, lipids, sugars, and salts [23]. While blue colors exist in nature, replicating them in foods can be challenging due to the low pH, light, and heat sensitivity of natural compounds producing blue tones [24]. PBPs have applications as food colorants and are found in various food-related applications, like C-phycocyanin (C-PC) which is a natural blue compound extensively explored and is the closest alternative to the dye Brilliant Blue FCF. In the year 2013, the FDA has approved PC from *Spirulina* to be used as a food colorant; it is currently used as a blue dye in various products, but its application in foods and beverages may be limited by its stability under heat, light, and acidic conditions, which can cause a loss of the blue color, especially during heat pasteurization or light exposure in beverages [24]. These issues create a particular challenge for the use of phycocyanin in beverage applications, where color stability is important for consumer preferences [24].

As an alternative to traditional heat pasteurization methods, high-pressure processing (HPP) can be used to pasteurize beverages at low temperatures using high hydraulic pressure (400–600 MPa) that inactivates microbes and extends the shelf life of beverages. This technique does not affect the structure of the chromophore in phycocyanin. Despite concerns about stability, C-PC demonstrates greater versatility compared to dyes like gardenia blue and indigo. It imparts a vivid blue color to jelly gum, soft candy, ice sherbets, popsicles, soft drinks, dairy items, wasabi, and ice creams. It is estimated that its annual market is in the range of USD 5–10 million. Dainippon Ink and Chemicals (Sakura, Japan) and Cyanotech Corporation, (Kailua-Kona, HI, USA) have already introduced this colorant to the market; it is commonly commercialized based on its purity grade, which is determined by the ratio of peak absorbance at 620 nm to protein absorbance at 280 nm. A ratio of ≥0.7 is considered food-grade purity. Moreover, C-PC offers antioxidant, anticancer, and anti-inflammatory effects, as documented in several studies [19,20,25,26,27]. In addition to blue dye, the marine red alga *Porphyridium* sp. is utilized to generate significant quantities of phycoerythrin (PE), which serves as a pink dye in gelatin products, ice cream, desserts, and confectionery items. PE exhibits a vibrant yellow fluorescence when exposed to ultraviolet light, making it suitable for enhancing the aesthetics of foods like candies, lollipops, soft drinks, and ice cream. Moreover, both pure PC and PE find applications in the cosmetic industry for crafting colorful, auto-fluorescent lipsticks, vivid eyeliners, and nail polish [19].

### 4.3. Medical Imaging

Phycobiliproteins are utilized in medical diagnostics as a fluorescent detection method across a range of areas concerning the diagnosis of different human conditions. They are applied in diagnosing human metabolic disorders, infectious diseases, cancers, and immune disorders [28]. Additionally, their use extends to diagnosing various infectious diseases in birds and mammals that could potentially threaten human health [29]. Moreover, these pigments are utilized in a variety of biological diagnostic studies, covering areas such as protein analysis, genomics, identifying plant transformants, detecting microbes, assessing food quality, and examining environmental factors. The utilization of PBPs as a detection system in diagnosing numerous metabolic disorders in humans is comprehensive [29]. For instance, the functional capacity of platelets in thrombosis and hemostasis can be assessed by examining the expression of Integrin αIIbβ3, which is typically low in resting platelets. This diagnostic approach involves employing R-phycoerythrin linked to a monoclonal antibody (mAb) targeting Integrin αIIbβ3. Alzheimer’s disease diagnosis relies on detecting the amyloid protein (Aβ) using the fluorochrome phycoerythrin, as amyloid constitutes the primary protein component of senile plaques in this condition. Similarly, the downregulation of CD5 expression on activated CD8+ T cells in hemophagocytic lymph histiocytosis is identified through an immunofluorescence kit containing the corresponding antibody conjugated with phycoerythrin. The use of PBPs in diagnosing human infectious diseases involves various methods. For instance, detecting the foodborne pathogen *E. coli* O157:H7 employs polyclonal antibodies linked to R-phycoerythrin. Additionally, immunotyping human papillomavirus can be achieved by employing monoclonal antibody-bound phycoerythrin in Luminex microspheres through a multiplexed assay system. PBPs serve as a crucial component in detecting a wide range of human cancers. This includes various types such as chronic lymphocytic leukemia, which is identified through CD5– FITC/CD19-phycoerythrin immunostaining. Liver cancer detection involves utilizing α-fetoprotein and carcinoembryonic antigen via a recombinant fusion protein, streptavidin-phycobiliproteins (SA-PBPs), in Sandwich ELISA. Human breast cancer is diagnosed using fluorescein isothiocyanate-conjugated anti-human leukocytic antigen-antibody and phycoerythrin-conjugated anti-mouse pan-leukocyte CD45 antibody. Colorectal cancer is identified with fluorescently labeled phycoerythrin antibodies targeting specific cell types. Leukemic B cell chronic lymphoproliferative disorders are differentiated through quantitative flow cytometry employing phycoerythrin-conjugated CD19, CD20, CD22, CD23, CD79b, and CD5 monoclonal antibodies, allowing discrimination between various types of leukemias and lymphomas. Detection extends to cancers affecting specific organs such as the pancreas, liver, colorectum, oral and nasopharyngeal regions, lungs, bones, ovaries, cervix, and prostate, as well as blood-related cancers like leukemia, lymphoma, and myeloma. Moreover, numerous follow-up cancer assessments have been conducted and patented [30].

### 4.4. Environmental Monitoring

Cyanobacteria, along with factors like chlorophyll-a levels, water clarity, and phosphorus content, are monitored to evaluate the ecological health of water bodies. This surveillance is crucial because harmful cyanobacteria pose a threat to global water sources, potentially contaminating drinking water. Under sufficient sunlight, these organisms can form dense, toxic blooms that produce cyanotoxins, leading to various health issues in humans, ranging from mild to severe reactions. Consequently, many water authorities have established alert levels for cyanobacterial presence and thresholds for cyanotoxin concentrations in both drinking water and recreational areas to safeguard public health. Detecting and managing dense cyanobacterial blooms can be challenging for water treatment facilities if they cannot effectively remove cyanotoxins. Traditional laboratory techniques such as taxonomic analysis, pigment extraction, and cyanotoxin testing are commonly used for cyanobacterial monitoring, but they are expensive, time-consuming, and may not capture rapid changes in water quality or sudden increases in cyanobacterial abundance. Phycocyanin, the predominant pigment found in cyanobacteria, can be isolated and measured because it absorbs light around 620 nm and emits fluorescence near 650 nm. Since cyanobacteria are the primary microorganisms in freshwater bodies that produce phycocyanin, high levels of this pigment suggest the presence of blooms. In vivo fluorescence from phycocyanin offers a promising alternative for online monitoring, as water quality in recreational areas and drinking water sources can be quickly assessed. Various commercially available submersible probes utilize in vivo fluorescence to monitor phytoplankton abundance, with some specifically targeting cyanobacteria. However, it is essential to validate any in vivo fluorescence monitoring probe before its use as a reliable tool for cyanobacterial surveillance. Additionally, remote sensing provides another means of monitoring water quality by collecting data from satellite sensors, which can be processed to quantify phycocyanin levels and serve as a proxy for cyanobacterial abundance in inland waters [20,31,32].

### 4.5. Veterinary Industry

Animal health is largely determined by its veterinary importance, as animals’ well-being is directly linked to food industries, to name just one example. This has driven research aimed at observing the biological effects of phycobiliproteins on animals [33]. For instance, extracts of phycocyanin derived from *A. platensis* were applied to poultry (broiler chickens); two groups were supplemented with 1 g/kg of body weight (T1) and 2 g/kg of body weight (T2), while the negative control group did not receive supplementation [34]. When comparing treatments, total protein and serum albumin levels increased, as did antioxidant enzyme activity, while serum cholesterol levels decreased in both treatments. Similarly, in broiler chickens, the use of microalgae species with high protein content (*Chlorella* sp.) improved daily weight gain and overall growth performance, which has been attributed to cellular remodeling induced by secondary metabolites and bioactive compounds [35].

PC extracts contain additional value-added molecules, which is why they are sometimes referred to as a “symbiotic product” (including polysaccharides, bioactive compounds, and proteins, among others). When these extracts were used in Ross broiler chickens, they were found to enhance meat redness and reduce levels of thiobarbituric acid-reactive substances. Additionally, they improved intestinal health without affecting growth performance [36]. Other studies further support these findings, such as the work of Omar et al. (2022) [37], which reported various biological effects in broiler chickens following the consumption of phycocyanin from *Spirulina platensis* at doses ranging from 0.25–1 g/kg/day over 35 days. It was observed that supplemented chickens had increased body weight and weight gain; however, as supplementation increased, feed conversion efficiency decreased linearly. Regarding blood parameters, total cholesterol and LDL levels decreased in groups receiving 0.25 and 1 g daily, while triglyceride levels decreased in groups receiving 0.25, 0.75, and 1 g. Antioxidant activity increased with higher supplement concentrations.

Other studies have investigated the effects of microalgae and their components on reproductive processes. For example, Wang et al. [38] evaluated the effects of phycocyanin on the reproductive efficiency of goats. They found that supplementation with 30 µg/mL improved oocyte polar body extrusion, cumulus expansion index, and parthenogenetic blastocyst formation compared to control and other concentrations (3, 10, 100, and 300 µg/mL). Furthermore, the effects on cryopreservation were also assessed, revealing that this concentration favored better cell morphology preservation and survival.

Considering the evidence and the reviewed literature, the use of phycobiliproteins as a supplement and their effects in veterinary medicine remain underexplored. However, biomass derived from microalgae has demonstrated positive effects on various aspects of animal health, suggesting that some responses are associated with protein consumption from this nutrient source. In poultry, studies on Lohmann Brown laying hens have shown beneficial effects after supplementation with *Chlorella vulgaris* and *Spirulina platensis*, combined with a conventional diet. This supplementation increased antioxidant capacity and elevated fatty acids, cholesterol, and β-carotenes in egg yolks [39].

In ruminants, the consumption of species such as *S. platensis* and *C. pyrenoidosa* has improved average daily weight gain in weaned steers during dry seasons [40]. Similarly, *Chlorella* sp. has proven to be an effective supplement for weight gain in calves, which increased their weight by up to 25 kg compared to 13.4 kg in non-supplemented calves. This improvement was linked to greater nitrogen assimilation, suggesting that the protein content of the algae played a role [41]. In cattle, supplementation with *Spirulina* sp. has been found to reduce somatic cells in milk, thereby increasing milk safety, as well as enhancing protein, fat, and lactose content in milk [42].

Not only have farm animals exhibited positive effects following supplementation with microalgae and/or their protein components, but evidence also suggests that *Spirulina* sp. consumption in dogs improves immune responses, as demonstrated by increased fecal IgA levels and more effective vaccination responses [43]. Similarly, some fish species benefit from microalgae consumption. For example, *Spirulina* sp. has been shown to enhance immune performance in carp by increasing white blood cell activity and IgM levels. In Nile tilapia, it has been found to promote body weight gain and antioxidant capacity [44,45]. It is important to highlight that, based on the reviewed literature, research on exclusive phycobiliprotein supplementation remains an area with great potential for generating new information, particularly concerning its veterinary relevance.

**Figure 3:**
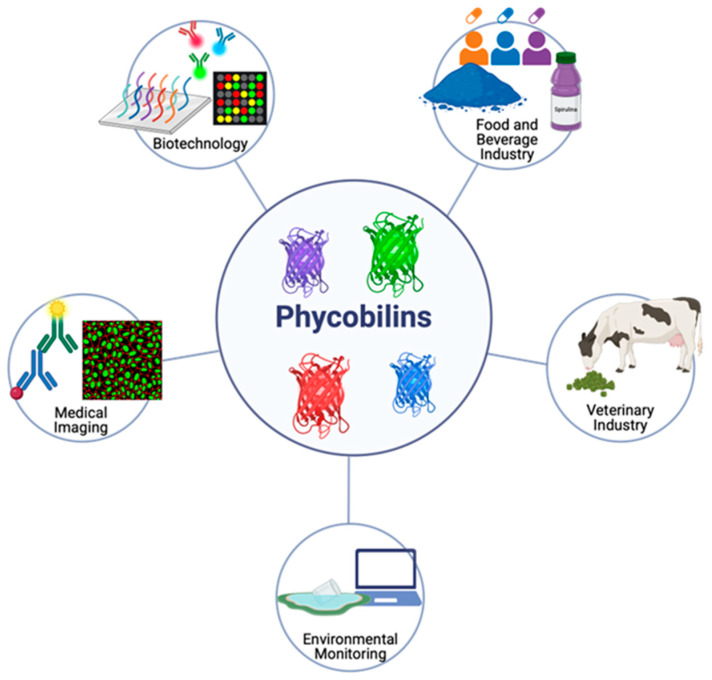
Industrial applications of Phycobilins (PBs).

In summary, phycobilins (PBs) have demonstrated remarkable potential as versatile biocomponents across various industrial sectors, including biotechnology, the food and beverage industry, medical imaging, environmental monitoring, and the veterinary field. These applications underscore their added value as natural pigments, functional agents, and diagnostic tools, contributing to the development of more sustainable, efficient, and safe solutions. Figure 3 provides a visual summary of these diverse application areas, highlighting the multifunctional role of PBs in modern industry.

## 5. Bioactivities

### 5.1. Therapeutic Agents

According to their spectral properties, phycobiliproteins (PBPs) can be classified into four groups: phycocyanin (PC), allophycocyanin (APC), phycoerythrin (PE), and phycoerythrocyanin (PEC). In recent years, the pharmaceutical properties of these proteins have been studied [46], enabling the various benefits provided by these biomolecules to be elucidated (Table 1).

**Figure 4:**
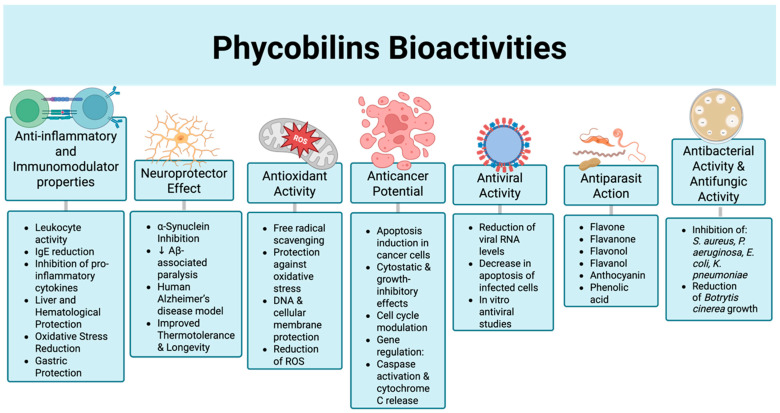
**Phycobilins** (PBs) bioactivities.

Among these benefits are antitumor properties, antioxidant activity, hepatoprotective and neuroprotective effects, among others (Figure 4).

### 5.2. Functional Properties of PBPs

#### 5.2.1. Anti-Inflammatory and Immunomodulator Properties

PBs, a class of light-harvesting bilins covalently bound to phycobiliproteins, are demonstrating promise as neuroprotective agents, prompting extensive research into their potential therapeutic applications [55]. These unique water-soluble pigments, predominantly derived from cyanobacteria and red algae, have garnered substantial interest from the biomedical research community due to their broad spectrum of biological activities, encompassing potent antioxidant and anti-inflammatory properties, in addition to their noteworthy neuroprotective effects [4,56].

PBPs, the pigment–protein complexes incorporating PBs, are naturally organized into phycobilisomes (PBSs), which are attached to thylakoid membranes. Beyond their established use as natural colorants in the food and cosmetics industries, PBs have also been examined to understand their antimicrobial, antioxidant, anti-inflammatory, and hepatoprotective properties, as well as their potential in clinical and immunological analysis [4,55].

The multifunctional nature of PBs and PBPs extends to various health-related applications, including immunomodulation, nephroprotection, and even photodynamic therapy [29]. Studies suggest C-PC is an effective inhibitor of the A53T (alanine 53 replaced with threonine) α-synuclein (αS) mutation, which causes the oligomerization of α-synuclein, reducing it by 50% at a ratio of 200:1 (αS:C-PC) [57]. The in vivo study conducted on *C. elegans* mutant strain CL4176 demonstrated a reduction in Aβ deposition and senile plaque formation following treatment with 100 μg/mL of PE. In *C. elegans*, thermotolerance was also improved, with survival extending to 20 ± 2.96 h compared to 18 ± 2.96 h in untreated worms [58]. Furthermore, APC moderated the expression of human beta-amyloid (Aβ1-42) and mitigated Aβ-associated paralysis in *C. elegans* CL4176 when exposed to elevated temperatures.

Phycoerythrin (PE) has demonstrated anti-inflammatory and Immunomodulator effects (Table 2) by inhibiting specific inflammatory markers, presenting its potential in managing inflammatory diseases [15,28]. The use of PBPs has been shown to influence the activity of the immune system. Mechanisms observed include changes in leukocyte activity, reduction of allergic responses through decreased IgE antibodies, and maintenance of mucosal function. These effects have been reported against various infectious diseases, particularly following the application of phycocyanin (PC) [59,60].

Regarding inflammatory processes, the effects of PC have been evaluated, demonstrating its ability to induce the production of proinflammatory molecules such as IL-6, IL-1β, and TNF-α. Additionally, a dose–response relationship has been observed, wherein PC promotes the production of proteins involved in phosphorylation events within signaling cascades associated with inflammatory molecules (e.g., COX-2, JNK, ERK). However, other studies have reported opposing effects following PC administration. Specifically, a dose–response relationship was noted in immunomodulatory activities, where PC reduced the production of proinflammatory molecules such as IFN-γ, as observed in mice models [61,62].

The C-PC of *S. platensis* showed a significant reduction in the elevated levels of hepatic enzymes alanine aminotransferase (ALT) and aspartate aminotransferase (AST), along with an improvement in endogenous antioxidant enzymes, including superoxide dismutase (SOD) and catalase (CAT), and a decrease in malondialdehyde (MDA) levels. Hematological analysis revealed statistically significant improvements in platelet counts and white blood cell levels in animals treated with C-PC and sodium arsenite compared to those treated with sodium arsenite alone. Furthermore, significant increases in hematocrit, hemoglobin, and erythrocyte levels were observed, indicating a comprehensive protective effect of C-PC against hematological and hepatic damage induced by arsenic [63]. Additionally, C-PC exhibits protective effects against ethanol-induced gastric ulcers in rats. Treatment at 50 mg/kg for five weeks increased serum hydroxyproline levels and gastric levels of HSP 70 (heat shock protein 70) and NP-SH (non-protein sulfhydryl compounds) [64]. Pro-inflammatory cytokine levels, such as NF-α (tumor necrosis factor alpha) and IFN-γ (interferon-gamma), were significantly reduced at 500 and 1000 mg/kg C-PC, while the anti-inflammatory cytokine IL-10 decreased in a dose-dependent manner at 100, 200, 500, and 1000 mg/kg [62].

Refs. [16,48,65] C-PC suppresses oxidative stress, inflammation, and apoptosis, and increases the viability of epithelial cells in lung tissues. It was demonstrated that C-PC inhibits the activation of the nuclear factor-kappa B (NF-κB)/NOD-like receptor protein 3 (NLRP3) pathway and the formation of the NLRP3 inflammasome complex [66]. C-PC suppressed biochemical markers of glycation, such as serum carboxymethyl-lysine (CML), and maintained the redox state by reducing lipid peroxidation and carbonyl content in proteins (CC), improving high-density lipoprotein cholesterol (HDL-C) activity and renal antioxidant enzyme activity [67].

**Table 2:** Anti-inflammatory and Immunomodulator effects of PBPs.

Organism	Assay Results	Reference
Phycocyanin (PC)		
*Spirulina platensis*	Hepatoprotective effect against carbon tetrachloride (CCl_4_)-induced toxicity in Wistar rats. A dose-dependent reduction in Alanine Transaminase (ALT) and Aspartate Transaminase (AST) levels was observed at 100, 150, and 200 mg/kg over 28 days. Histological analysis revealed a recovery comparable to the negative control.	[68]
*S. platensis*	The administration of C-PC to Wistar rats at doses of 250 and 500 mg/kg over 28 days did not induce toxicity or cause changes in organ weights or in biochemical or hematological profiles; it did not result in damage to the heart, lungs, liver, stomach, kidneys, uterus or testes.	[69]
*Phormidium versicolor* NCC466	Treatment with 50 mg/kg of C-PC reduced ALT, AST, and bilirubin levels in a cadmium (Cd)-induced liver injury model in Wistar rats.	[70]
N/D	It reduced serum malondialdehyde (MDA) levels and gastric pro-inflammatory markers, including TNF-α, IL-1β, IL-6, ICAM-1, and MPO (myeloperoxidase).	[64]
*Spirulina* sp.	Administration of C-PC to C57BL/6 mice significantly reduced plasma levels of alanine aminotransferase (ALT) and aspartate aminotransferase (AST). Histological analysis confirmed protection against radiation-induced hepatotoxicity. The treatment increased the relative mRNA expression of superoxide dismutase (SOD) and glutathione peroxidase (GSH-PX) while reducing ROS levels in the liver. Furthermore, H2AX expression (a marker of DNA damage) was markedly lower compared to the irradiated group. Also, can inhibit the formation of Aβ40/42 fibrils.	[57,71]
*S. platensis*	C-PC exhibited antidiabetic activity with IC_50_ values of 231.45 ± 0.47 μg/mL for α-amylase and 198.11 ± 0.25 μg/mL for β-glucosidase. These values were compared to the standard control, acarbose, which showed IC_50_ values of 151.96 ± 0.57 μg/mL and 141.33 ± 0.34 μg/mL, respectively. Additionally, C-PC demonstrated dose-dependent anti-inflammatory activity, inhibiting proteinase activity by 5, 11, 17, 29, and 36% at concentrations of 100, 200, 300, 400, and 500 μg/mL, respectively.	[72]
*S. platensis*	The administration of C-PC at a dose of 300 mg/kg in albino rats demonstrated a protective effect against arsenic-induced toxicity.	[63]
*Mycrocystis aeruginosa*	C-PC (50 mg/kg) exhibits a therapeutic renal effect against potassium dichromate (PD)-induced kidney injury in Wistar rats. This is achieved through improved renal function, downregulation of oxidative stress and the TLR4/TNF-α/HSP70 inflammatory pathway, as well as modulation of IGF-1.	[73]
N/D	C-PC was determined to be non-toxic at a concentration of 2000 mg/kg in Balb/c mice. Serum concentrations of SOD and CAT remained comparable to the vitamin E control at a dose of 200 mg/kg. SOD and CAT levels exhibited a dose-dependent increase at 500 and 1000 mg/kg of C-PC.	[62]
N/D	C-PC exhibits neuroprotective effects against chemotherapy-induced cognitive impairment (CICI), commonly known as “chemo brain”. Treatment with 50 mg/kg significantly improved the behavioral deficits of mice treated with doxorubicin (DOX). Additionally, it suppressed DOX-induced neuroinflammation and oxidative stress, mitigated mitochondrial abnormalities, rescued dendritic spine loss, and increased synaptic density in the hippocampus of DOX-treated mice.	[74]
N/D	C-PC is not cytotoxic to macrophages (RAW 264.7). It promotes proliferation with a relative survival of 138% at 200 μg/mL. It reduces nitric oxide (NO) levels by 39% and 41.16% at concentrations of 50 and 200 μg/mL, respectively. It exhibits inhibitory effects on TNF-α by 74.32% and 100% at concentrations of 50 and 200 μg/mL. It also inhibits IL-6 by 30.44% and 75.76% at 50 and 200 μg/mL, respectively. At a dose of 30 μg/mL, phycocyanin shows higher inhibition of Collagen I (an indicator of idiopathic pulmonary fibrosis) and promotes cell recovery in the A549 cell line.	[75]
*P. versicolor* NCC466	The nephroprotective activity against cadmium (Cd) toxicity induced by 35 μg of Cd in HEK 293 cells was evaluated. Cells treated with 25 μg of C-PC increased cell viability by 90%. Antioxidant enzymes catalase (CAT), superoxide dismutase (SOD), and glutathione peroxidase (GSH-Px) in the kidneys of rats treated with C-PC significantly counteracted the prooxidant effect of Cd exposure.	[76]
*Plectonema* sp.	The administration of C-PC in diabetic Wistar rats for 45 days decreased triglyceride (TG) levels, blood glucose, glycated hemoglobin (HbA1c), total cholesterol (TC), low-density lipoprotein cholesterol (LDL-C), liver and kidney function indices, and increased body weight in diabetic rats.	[67]
*S. platensis*	In a colorectal cancer-associated colitis model induced by azoxymethane (AOM) and dextran sulfate sodium (DSS) in C57BL/6J mice, C-PC improved inflammation induced by AOM/DSS. Treatment with C-PC significantly reduced the number of colorectal tumors and inhibited the proliferation of epithelial cells in CAC mice.	[77]
N/D	C-PC significantly alleviates pathological damage in an acute lung injury (ALI) model in C57BL/6 mice induced by seawater (25%) and lipopolysaccharide (5 mg/kg).	[66]
Phycoerythrin (PE)		
*Colaconema formosanum*	Non-toxic to NIH-3T3 embryonic fibroblasts cells, showing viability of 92.1 ± 1.03, 90.0 ± 1.11, 89.2 ± 1.41, and 82.4 ± 1.21% in a dose-dependent manner at 1, 2, 5, and 10 μg/mL, respectively. Exhibited anti-allergic activity by arresting β-hexosaminidase release (82.4% at 20 μg/mL). Promoted type I procollagen synthesis, with 10 μg/mL generating 130.7 ± 4.2% compared to the TGF-β1 control (121.3 ± 4.6%).	[78]
*Lyngbya* sp. A09DM	Reduction in Aβ deposition on *C. elegans* mutant strain CL4176.	[79]
Allophycocyanin (APC)		
*Phormidium* sp. A09DM	In wild-type *C. elegans*, treatment with 100 μg/mL of APC extended the lifespan from 16 ± 0.2 days (control) to 20 ± 0.1 days.	[58]

PBPs: Phycobiliproteins, C-PC: C-Phycocyanin, N/D: Not Described, AST: Aspartate Transaminase, ALT: Alanine transaminase, TNF-α: Tumour Necrosis Factor alpha, IL-1β: Interleukin-1-beta, IL-6: Interleukin-6, ICAM-1: Intercellular Adhesion Molecule-1, H2AFX: H2A Histone Family Member X, TLR4:Toll-like receptor 4, HSP70: Heat shock protein 70, IGF-1: Insulin-like Growth Factor 1, SOD: Superoxide Dismutase, Cd: Cadmium, CAT: Catalase, CAC: Colitis-Associated Cancer, TGF-β1:Transforming Growth Factor Beta 1, ROS: Reactive Oxygen Species, mRNA: Messenger RNA, DNA: Deoxyribonucleic Acid, IC_50_: Half Maximal Inhibitory Concentration, CCl_4_: Carbon Tetrachloride, MDA: Malondialdehyde, MPO: Myeloperoxidase, GSH-Px: Glutathione Peroxidase, PD: Potassium Dichromate, CICI: Chemotherapy-Induced Cognitive Impairment, DOX: Doxorubicin, NO: Nitric Oxide, TG: Triglyceride, TC: Total cholesterol, LDL-C: Low-Density Lipoprotein Cholesterol, AOM: Azoxymethane, DSS: Dextran Sulfate Sodium, ALI: Acute Lung Injury, Aβ: Amyloid beta peptide, *C. elegans*: *Caenorhabditis elegans*.

#### 5.2.2. Antioxidant Activity

PC displays strong antioxidant activity (Table 3), scavenging free radicals and protecting cells from oxidative damage, which could benefit overall health [29,80]. Adverse effects caused by oxidative stress resulting from daily metabolic processes have been widely reported. Such evidence highlights damage to DNA and cellular membranes, including their lipid and protein components [81]. However, various studies also indicate the potential advantages of certain molecules against oxidative stress. Li (2022) demonstrated that phycobiliproteins from *S. platensis* exhibit protective effects on DNA, preventing damage induced by oxidizing agents such as peroxynitrite [82].

In addition, PBPs have been shown to reduce the effects of reactive oxygen species (ROS), exhibiting comparable effectiveness to other phytochemicals. This activity supports protection against inflammation, cardiovascular damage, diabetes, degenerative injuries, and cancer, all of which may be triggered by ROS [5,47]. Complementing this, evidence suggests that phycobiliproteins promote the production of antioxidant enzymes [83]. Among the elucidated mechanisms supporting this biological activity are their ability to “scavenge” hydroxyl and alkoxyl radicals in in vitro assays. Moreover, they demonstrate reduced microsomal lipid peroxidation in human polymorphonuclear leukocytes activated by zymosan [48,84].

Furthermore, hydrolysates of PBPs from *A. platensis*, generated using enzymes such as trypsin, papain, and bromelain, have been evaluated. These hydrolysates yield peptides with biological activity, providing potential applications for nutritional supplementation and health [85]. This is consistent with the findings of Sonani et al. (2014) [86], who reported significant antioxidant activity of phycoerythrin (PE), allophycocyanin (APC), and phycocyanin (PC) from cyanobacteria of the genus *Lyngbya*. Among these, PE exhibited the highest antioxidant activity, while APC showed the lowest. Nevertheless, the pharmaceutical potential of PE remains insufficiently explored [86].

The aforementioned PBPs have been reported to exhibit antioxidant activity in in vitro studies. PC derived from microalgae of the genera *Spirulina, Arthrospira,* and *Aphanizomenon*, among others, have demonstrated notable antioxidant properties [48,84,87,88]. Furthermore, various pathological conditions have been linked to the oxidative activity of reactive oxygen species (ROS). It has been shown that PCs can mitigate the detrimental effects of ROS, including liver damage, neurotoxicity, and renal oxidative stress. Additionally, PCs have demonstrated protective effects in renal tissue and exhibit anti-tumor activity. These observations have primarily been documented through in vitro experiments [89].

**Table 3:** Antioxidant effects of PBP.

Organism	Assay Results	Reference
Phycocyanin (PC)		
*Arthrospira platensis* IFRPD 1182	The C-PC obtained through lyophilization, and dehydration showed a DPPH activity of 58% and 58.6%, respectively, at a concentration of 0.06 g/mL.	[90]
*S. platensis*	The SC_50_ activity for DPPH was 104 μg/mL.	[68]
*S. platensis*	The DPPH activity showed a dose-dependent response with 25%, 57%, 76.2%, 97.2%, and 99% inhibition at concentrations of 10, 25, 50, 75, and 100 μg/mL, respectively.	[69]
*P. versicolor* NCC466	The C-PC showed a superoxide radical (O_2_^−^) and hydroxyl radical (OH) scavenging capacity of 87.42% and 88.75%, a nitric oxide (NO) radical antioxidant activity of 84.87%, and an Iron (II) chelation activity of 78.56%.	[70]
*S. platensis*	An SC_50_-DPPH activity of 18.59, 34.23, and 47.26 mg/mL was reported for a formulation of C-PC and maltodextrin at concentrations of 50, 100, and 150 g/L, respectively, with an SC_50_ control of 45.21 mg/mL for non-formulated C-PC.	[91]
*Arthrospira* sp.	The gelatin-C-PC formulation showed a dose-dependent Iron (II) chelation activity of 30%, 85%, and 92% for concentrations of 1, 2.5, and 5 mg/mL (C-PC), and a DPPH activity of 46.73%, 60%, 77%, and 78.9% for 0.5, 1, 2.5, and 5 mg/mL, respectively.	[92]
*Nostoc* sp. R76DM	In vitro, 100 μg/mL showed DPPH antioxidant activity of 63.15%, FRAP 0.22%, and reducing power of 0.40%. In vivo, it had a protective effect against oxidative stress of 84.04% in the *Caenorhabditis elegans* model.	[93]
*Oscillatoria minima*	At a concentration of 1 mg/mL, a DPPH activity of 44% and an ABTS activity of 95% were obtained, which was higher than the positive control Butilhidroxianisol (BHA at 90%).	[94]
*S. platensis* LEB-52	It exhibited an ABTS antioxidant activity of 161.66 ± 4.64 μmol TE.g^−1^ and an oxygen radical absorbance capacity (ORAC) of 1211.41 ± 73.65 μmol TE.g^−1^.	[95]
*A. platensis*	The purified extract showed a DPPH activity of 98% at 25 μg/mL and an Iron (II) chelation activity of 100% at 5 μg/mL.	[96]
*Galdieria sulphuraria*	The ABTS antioxidant activity of the phycocyanins obtained from the algae *G. sulphuraria* and *S. platensis* at 1 g/mL was 72.97% and 75.55%, respectively.	[97]
N/D	The IC_50_ of DPPH activity was 158.3 μg/mL (control 112.9 μg/mL), ferric reducing antioxidant power (FRAP) was 152.7 μg/mL (control 91.47 μg/mL), hydroxyl radical scavenging was 88.67 μg/mL (control 57.78 μg/mL), hydrogen peroxide scavenging was 110.9 μg/mL (control 44.63 μg/mL), and total antioxidant capacity (TAC) was 164.78 μg/mL (control 26.76 μg/mL). Ascorbic acid was used as the control.	[98]
*S. platensis* MK343101	The antioxidant activity against peroxide radicals was 97.7% at 100 μg/mL.	[99]
*Pseudanabaena* sp. ABRG5-3, *Limnothrix* sp. SK1-2-1 *y A. platensis* NIES-39	The C-PC obtained from the three strains showed an ABTS antioxidant activity above 80% at a concentration of 1 mg/mL.	[100]
*Porphyra* sp.	DPPH antioxidant activity showed a dose-dependent effect, with 23.87 ± 1.12% at 5 mg/mL and 59.46 ± 1.23% at 10 mg/mL. Iron (II) chelation activity was 67.10 ± 0.45%, 73.61 ± 0.31%, 86.18 ± 0.30%, and 90.31 ± 0.11% at concentrations of 31.25, 62.50, 125, and 250 μg/mL, respectively.	[101]
*S. platensis*	It exhibited a DPPH antioxidant activity of 94.284% at 300 mg/mL.	[63]
*S. platensis*	An antioxidant activity of 87% was reported for DPPH.	[102]
*Plectonema* sp.	The DPPH antioxidant activity was 58.75%, nitric oxide (NO) radical scavenging activity was 58.4%, and peroxide radical (O_2_^−2^) scavenging capacity was 61.5%. These data were obtained at a concentration of 1000 μg/mL.	[103]
*A. platensis*	The ORAC was 12,141 ± 1928 and 32,680 ± 3295 TE/100 g for commercial and isolated C-PC, respectively.	[104]
*A. platensis*	An IC_50_ value of 629.94 μg/mL for DPPH was obtained. It showed an anti-inflammatory activity of 74.49% and an anti-arthritis activity of 76.98%.	[6]
*Caquena* (CAQ-15)	PC (CAQ-15) showed ABTS and FRAP activities of 312 ± 15 and 1.55 ± 0.10 μmol TE/100 mg, respectively. PC (LLA-10) exhibited ABTS and FRAP activities of 205 ± 41 and 2.50 ± 0.15 μmol TE/100 mg, respectively.	[105]
Phycoerythrin (PE)		
*Palmaria* sp.	An antioxidant activity of 21.3% for DPPH and 90.4% for ABTS was obtained at concentrations of 5 mg/mL and 1 mg/L of PE, respectively.	[106]
*Spyridia filamentosa*	It showed IC_50_ antioxidant activity in various assays: DPPH of 125.5 μg/mL, nitric oxide of 87.85 μg/mL, hydroxyl radical of 34.56 μg/mL, superoxide radical (O_2_) of 18.58 μg/mL, and ABTS of 3.13 μg/mL.	[107]
*Kappaphycus alvarezii*	An IC_50_ DPPH activity of 31.02 μg/mL was reported.	[108]
*Nostoc* sp.	PE exhibited ABTS and FRAP antioxidant activities of 198 ± 45 and 0.92 ± 0.15 μmol TE/100 mg, respectively.	[105]
*Nostoc* sp. A5	The DPPH antioxidant activity showed an IC_50_ of 0.038 mg/mL, with ascorbic acid as the control at 0.032 mg/mL. The ABTS activity had an IC_50_ of 0.02 mg/mL, with butylhydroxytoluene (BHT) as the control at 0.019 mg/mL. The superoxide radical antioxidant activity had an IC_50_ of 0.057 mg/mL (PE) and 0.042 mg/mL (ascorbic acid). A dose-dependent inhibition of nitric oxide was observed, with inhibition percentages of 79, 84, 91, and 98% for 10, 25, 50, 100, 150, and 250 μL, respectively.	[109]
*Porphyra* sp.	The DPPH antioxidant activity showed a dose-dependent effect, with 30.17 ± 0.95% and 63.06 ± 1.08% inhibition at 5 and 10 mg/mL, respectively. The iron (II) chelation activity was 60.59 ± 0.74%, 71.61 ± 0.52%, 87.63 ± 0.23%, and 91.21 ± 0.11% at 31.25, 62.50, 125, and 250 μg/mL, respectively.	[101]
*Nostoc* sp.	Both strains exhibited antioxidant activity with an IC_50_ of 0.03 mg/mL for DPPH and 0.04 mg/mL for ABTS.	[110]
*Gracilaria corticata*	Effective activity in antioxidant capacity (264.90 ± 10.20 μg/mL), DPPH antioxidant activity (22.91 ± 1.90% at 0.15 mg/mL), and iron (II) chelation effect (26.06 ± 1.60% at 0.15 mg/mL).	[111]
Allophycocyanin (APC)		
*Corallina officinalis*	An IC_50_ DPPH activity of 893.39 μg/mL was obtained. It exhibited an anti-inflammatory activity of 74.81% and an anti-arthritis activity of 78.25%.	[6]

PBPs: Phycobiliproteins, C-PC: C-Phycocyanin, DPPH: 2,2-diphenyl-1-picrylhydrazyl, SC_50_: Half Scavenging Concentration, FRAP: Ferric Reducing Antioxidant Power, ABTS: 2,2′-azino-bis(3-ethylbenzothiazoline-6-sulfonic acid), ORAC: Oxygen Radical Absorbance Capacity, IC_50_: Half Maximal Inhibitory Concentration, TE: Trolox Equivalent, N/D: Not Described. O_2_: Superoxide Radical, OH: Hydroxyl Radical, NO: Nitric Oxide, BHA: Butilhidroxianisol, TAC: Total Antioxidant Capacity, BHT: Butylhydroxytoluene.

#### 5.2.3. Mechanisms of Antioxidant and Anti-Inflammatory Activity of PBPs

PBPs, particularly phycocyanin (PC) and phycoerythrin (PE), exert their antioxidant effects primarily through direct scavenging of reactive oxygen species (ROS), including superoxide anions, hydroxyl radicals, and hydrogen peroxide. These proteins also modulate the expression of antioxidant enzymes, such as superoxide dismutase (SOD), catalase (CAT), and glutathione peroxidase (GPx), by activating transcription factors like Nrf2 (nuclear factor erythroid 2-related factor 2). The activation of Nrf2 facilitates the cellular defense response against oxidative stress by enhancing antioxidant gene expression [112,113].

On the anti-inflammatory front, PBPs have been shown to inhibit the activation of NF-κB (nuclear factor kappa-light-chain-enhancer of activated B cells), a key regulator of pro-inflammatory cytokine production [114,115]. As a result, levels of TNF-α, IL-6, and IL-1β are significantly reduced in inflammatory models [16,116]. PBPs also interfere with the MAPK signaling pathway, particularly the ERK, JNK, and p38 kinases, further contributing to their anti-inflammatory potential [117]. In macrophages and other immune cells, PBPs modulate the release of nitric oxide (NO) and prostaglandin E2 (PGE2) through downregulation of iNOS and COX-2 expression [87].

**Figure 5:**
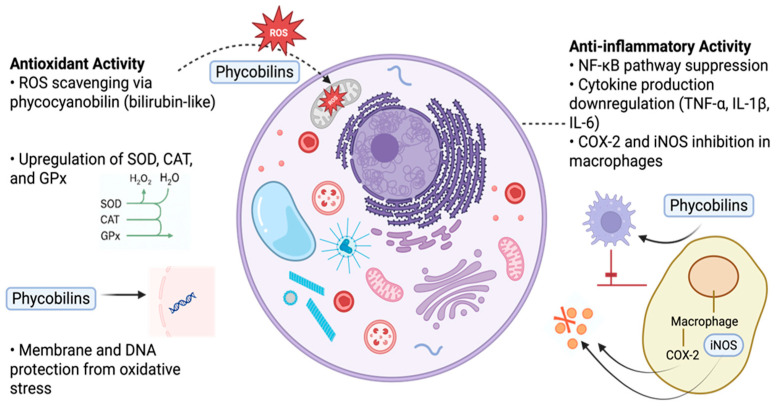
Molecular pathways involved in the antioxidant and anti-inflammatory effects of phycobilins in eukaryotic cells [82,118].

These mechanisms suggest that PBPs play a dual protective role by limiting oxidative damage and modulating immune responses, which positions them as promising candidates for the development of anti-inflammatory and antioxidant therapies [114,116]. As illustrated in Figure 5, PBs interact with various cellular signaling pathways to exert their biological effects [82,118]. Their antioxidant activity is primarily mediated by scavenging reactive oxygen species (ROS) and enhancing endogenous antioxidant defenses, while their anti-inflammatory role involves downregulation of pro-inflammatory mediators such as TNF-α, IL-6, and COX-2, often through the inhibition of NF-κB signaling [16,48,65]. These mechanisms position PBs as promising natural compounds for therapeutic and nutraceutical applications [119].

### 5.3. Anticancer Potential

In vitro studies suggest that PBs, such as allophycocyanin, have anticancer potential by inducing apoptosis in cancer cells, highlighting their promise as cancer therapeutics [15,120]. Evidence indicates that PCs possess cytostatic capacity and growth-inhibitory effects on colon cancer cells (HT-29) and lung cancer cells (A549). Additional evidence highlights the use of PEs, which can inhibit the growth of HeLa cells, achieving inhibition rates ranging from 37.3% to 62%; notably, the source of this protein is heterologous. Similarly, other heterologous PBPs, such as APCs, have demonstrated inhibitory potential against liver cancer cells (H22), with inhibition rates ranging from 36% to 62% [81,121,122].

Furthermore, according to Noore et al. (2023) [123], a crude extract of PBPs derived from *Porphyridium purpureum* exhibited high cytotoxicity against the colorectal adenocarcinoma cell line Caco-2. This extract was characterized by the presence of PE, PC, and APC. In relation to the aforementioned findings, nanotechnology has also been linked to the evaluation of PBPs’ biological activity [123]. For instance, Silva-Núñez et al. (2024) [124] reported that the encapsulation of PE with various materials can enhance its anticancer activity, significantly reducing the viability of colorectal cancer cells (Caco-2).

Among the proposed mechanisms, it has been suggested that PBPs can interfere with the cell cycle of cancerous cells, a biological process closely associated with the progression of this disease. As demonstrated in the study by Liu et al. (2000), phycocyanin (PC) derived from *S. platensis* inhibits the growth of blastomyelogenic leukemia cells by affecting the S phase and arresting the G1 phase of the cell cycle [125]. Similarly, PC from *Oscillatoria tenuis* has been shown to reduce the activity of the G2 and M phases in HT-29 and A549 cancer cell lines compared to the control. An increase in the percentage of cells in the G0/G1 phase was also observed, highlighting the potential of PBPs to modulate the cell cycle in tumorigenic contexts.

The process of apoptosis in cancer cells represents another biological mechanism targeted for therapeutic intervention, making studies focused on this alternative highly relevant. Subhashini et al. (2004) investigated the effect of phycocyanin (PC) on the K562 cell line (chronic myeloid leukemia), demonstrating that polymerase cleavage occurred and cytochrome C was released into the cytoplasm [126]. Additionally, the expression of apoptosis-related genes was analyzed, revealing a downregulation of the anti-apoptotic gene Bcl-2. However, pro-apoptotic genes, such as Bax, did not exhibit significant changes in expression. Moreover, studies have demonstrated that the antitumor effect of PBPs can be extrapolated to in vivo models [127]. For instance, the inhibitory activity of phycocyanin (PC), which follows a dose-dependent response, was demonstrated in a murine model induced with tumor development through TPA (tissue plasminogen activator) induction [128]. Additional evidence suggests that the interaction between PBPs and anticancer drugs may exhibit synergistic activity. This was shown in a study by Gantar et al. (2012), where the combination of PC with Topotecan demonstrated higher effectiveness compared to the individual application of the drug [129]. These findings underscore the potential of PBPs to enhance the efficacy of conventional anticancer treatments.

The search for alternatives to enhance the antitumoral activity of phycobiliproteins has directed scientific efforts toward the integration of various fields of knowledge. According to Rashed et al., (2023), the anticancer activity of phycocyanobilin (PCB) was evaluated against the colorectal cancer cell line HT-29. PCB is a molecule derived from the cleavage of phycocyanin from the entire protein structure [130]. It demonstrated anticancer activity by promoting the overexpression of the NME1 gene (anti-metastatic) and reducing the expression of the COX-2 gene. Additionally, this activity was significantly lower when compared to the performance of PCB conjugated to a metal-organic framework (UiO-66), which enhanced its antitumoral activity by up to twofold. This section highlights the potential of PC as a modulator of apoptosis pathways in cancer therapy (Table 4).

### 5.4. Antibacterial Activity & Antifungic Activity

The antimicrobial potential of phycobiliproteins suggests their utility in developing alternative therapies against antibiotic-resistant pathogens and fungal infections [4,29].

PCs have demonstrated bactericidal activity when integrated into silver nanoparticles, showing inhibition of the growth of *S. aureus*, *P. aeruginosa*, *E. coli*, and *K. pneumoniae*, all of which are considered medically important species [23]. Not only has antibacterial activity been observed, but antifungal activity has also been demonstrated, specifically a reduction in the growth of *Botrytis cinerea* [145].

On the other hand, studies involving the application of PE show various effects related to obesity. This protein promotes the growth of beneficial bacteria while simultaneously reducing the growth of harmful bacteria [33]. Considering other factors related to obesity, studies in animal models have shown that PE consumption lowers blood glucose and cholesterol levels, in addition to reducing food intake [29].

Further research is needed to optimize PBPs efficacy and establish their clinical applications. Table 5 is a list of some effects of PBPs against bacteria and fungus infections.

### 5.5. Antiviral Activity

Although some studies suggest the potential of phycobiliproteins as antiviral agents, direct evidence of their effects on viral activity remains limited. However, in vitro, in vivo, and in silico investigations have explored their possible applications against various viruses, highlighting their activity against non-enveloped viruses and coronaviruses [29]. Also has been reported that these proteins might have antagonistic effects against non-enveloped viruses [148]. Among the phycobiliproteins with antiviral activity, C-PC and APC stand out, both of which are found in high concentrations in various microalgae. Singh et al. (2023) [149] demonstrated that C-PC exhibits antiviral activity against the influenza H1N1 virus in in vivo models, improving survival rates.

Also, studies have shown that the application of APC inhibits the proliferation of enterovirus-71 viral particles and reduces apoptotic activity (through the quantification of viral RNA) in infected cells in in vitro studies [150]. C-PC from *Spirulina* sp. has demonstrated significant antiviral activity against human immunodeficiency virus (HIV)-1 strains (92/RW/008, Ada5, VB051, UG070) in the TZM-bl cell model. The compound exhibited a high safety profile, with CC_50_ values of 0.986 mg/mL for TZM-bl cells and 0.872 mg/mL for peripheral blood mononuclear cells (PBMC). Additionally, C-PC effectively inhibited HIV-1 replication, with IC_50_ values of 0.0846, 0.0817, 0.1603, and 0.1740 mg/mL against the respective HIV-1 strains. This inhibitory activity was further confirmed in PBMC-HIV-1 VB051 cells, where an IC_50_ of 0.09 mg/mL was observed [151].

Spirulina has demonstrated antiviral activity against SARS-CoV-2 by inhibiting the active site of NSP12, a key non-structural protein involved in viral RNA replication [148]. Additionally, Ismail et al. (2021) [152] designed silver nanoparticles functionalized with phycobiliproteins extracted from *Spirulina platensis* and *Nostoc linckia*. These nanoparticles exhibited significant antiviral activity compared to ribavirin (66.67%), with the *S. platensis*-based formulation showing 48.33% activity and the *N. linckia*-based formulation showing 64.97% activity.

Additionally, various studies have assessed the antiviral potential of phycobiliproteins through computational simulations. Munawaroh et al. (2024) [153] analyzed the effects of these compounds derived from *S. platensis* against severe acute respiratory syndrome coronavirus 2 (SARS-CoV-2), identifying binding affinities to three viral targets: (a) the main protease, (b) the receptor-binding domain, and (c) the RNA-dependent RNA polymerase. Phycoerythrobilin exhibited the highest affinity for the RNA polymerase and receptor-binding domain compared to the control (remdesivir), while phycocyanobilin showed lower affinity for the main protease than nelfinavir.

Studies combining in silico and in vitro approaches have also demonstrated the antiviral efficacy of these proteins. Jadaun et al. (2022) [151] evaluated C-PC against various HIV-1 strains, predicting its interaction with protease and reverse transcriptase through molecular simulations, results that were later confirmed in experimental assays. Similarly, Pendyala et al. (2021) [22] studied the antiviral activity of phycobiliproteins against SARS-CoV-2 using in silico models and in vitro tests. They observed that phycocyanobilin inhibits both the main protease and the papain-like protease. Additionally, in silico studies indicated that phycobiliproteins such as phycourobilin, phycoerythrobilin, and phycoviolobilin exhibit similar affinities toward these viral targets.

These findings support the hypothesis that phycobiliproteins could represent a promising alternative in the search for new antiviral agents. However, further research is needed to validate their efficacy in preclinical and clinical models.

## 6. Extraction and Purification Methods

The extraction and purification of phycobiliproteins (PBPs) from cyanobacteria and red algae are crucial steps that determine the yield, purity, and functional integrity of these valuable biomolecules [16,154]. These steps involve two distinct but complementary processes: extraction, which refers to the release of PBPs from the cell matrix, and purification, which is aimed at isolating and concentrating the PBPs with minimal contaminants [155,156].

### 6.1. Extraction Techniques of Phycobiliproteins

The extraction of phycobiliproteins from cyanobacteria or red algae involves disrupting the cell wall to release the intracellular contents. Mechanical methods such as sonication, bead milling, and freeze–thaw cycles are frequently used, offering varying efficiencies depending on the organism and target protein. Sonication is rapid and effective, though prolonged exposure may denature proteins. Bead milling achieves high yields but can generate significant heat, potentially degrading thermolabile compounds [154,157].

Non-mechanical methods, such as enzymatic lysis or solvent extraction using buffers, are milder and tend to preserve the structural integrity of PBPs. However, they are often slower and may be more expensive. Methods such as osmotic shock and enzymatic digestion have been employed to reduce damage to PBPs and preserve their bioactivity, although these are often slower and less scalable [156]. Recent advances include green extraction techniques like ultrasound-assisted extraction (UAE), supercritical fluid extraction (SFE), and the use of deep eutectic solvents (DESs). These methods are environmentally friendly and improve the sustainability of PBP recovery processes. UAE, for instance, enhances mass transfer and reduces solvent usage, while DESs offer low toxicity and customizable solvent systems [155,158].

**Figure 6:**
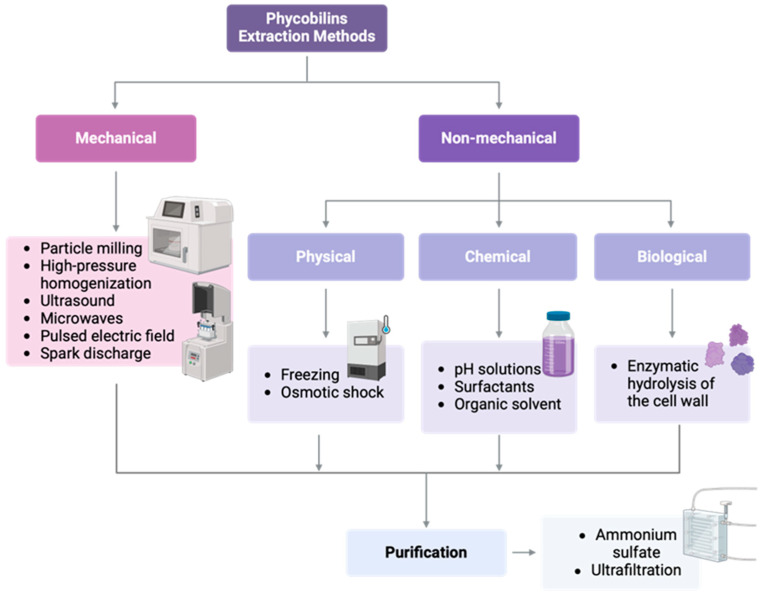
Extraction methods of PBs.

To liberate PBPs from cells, methods that destroy the cell’s structure and properties must be avoided. The process of separation may become complicated due to the solubility of various impurities present in cells, including polysaccharides that can be dissolved using specific techniques. Cell disruption techniques usually include both mechanical and non-mechanical methods. Mechanical methods include particle milling, high-pressure homogenization, ultrasound, and microwaves. Osmotic shock, enzymatic systems, chemical reagents, and repetitive cycles of freezing and unfreezing are examples of non-mechanical techniques (Figure 6).

By utilizing non-mechanical techniques, such as low temperatures or the addition of specific substances, cell walls can be ruptured. One common technique to induce cell wall rupture is freezing. After freezing at −20 °C, microalgae are thawed at −4 °C to breach the cell wall and release intracellular chemicals. Frequent freezing may increase efficiency by disrupting the cell wall [159]. In 2014, Zhao extracted phycocyanin from dried algae powders of *S. platensis* by freezing at −20 °C and, subsequently dissolving at 4 °C four times with distilled water procedures; a purity of 0.42 was obtained [160]. Osmotic shock is a direct extraction method that releases PBP through the manipulation of internal or external osmotic pressure after the immersion of microalgae in an extraction buffer for an extended duration in the absence of light or after their combination with distilled water [161]. *Arthrospira* (Spirulina) was employed to extract phosphate buffer phycocyanin with a yield of 8.66% by dry weight of the biomass. After this, purification methods were utilized, including ultrafiltration, final column chromatography, activated charcoal, Sephadex G100, and DEAE Sepharose Fast Flow. Alternatively, by modifying the buffer’s pH or adding chemical reagents to the sample solution, such as surfactants or organic solvents, cell walls can be broken down or intracellular materials liberated. The buffer’s pH level controls the protein’s electron charge properties, which can improve the product’s solubility. Researchers have found that using NaCl salts is a good way to get PBPs out of the dried biomass of *S. platensis*. An extraction rate of 74.8% was achieved, resulting in a yield of 102.4 mg/g of phycocyanin with a purity of 0.74 [162]. Enzymatic cell wall hydrolysis is thought to be a less aggressive and more ecologically friendly method. Bacterial enzymatic hydrolysis yielded concentrations of 0.36 and 0.86 mg/mL from phycoerythrin and phycocyanin extracts from *Porphyra* [101].

Mechanical techniques disrupt microalgal cells with physical force. For the liberation of PBPs, the microalgae are burned during the high-pressure treatment via the cutting and high pressure generated by the machine. While this approach ensures consistent treatment and generates minimal heat, it is crucial to consider the pressure tolerance of individual microalgae. Ultrasonic Assisted Extraction (UAE) is a technique for destroying the cell membranes of microalgae by passing a liquid medium through a high-frequency ultrasound that generates shock waves and excessive heat. To refine *A. platensis*, phycocyanin was extracted using an ultrasound bath-assisted extraction method utilizing reusable ionic liquid. The resulting phycocyanin had a yield of 0.85 mg/g and a purity of 1.54 [163]. Although microwave-assisted extraction can extract PBPs rapidly, thermal denaturation of PBPs can occur as time increases. Combined microwave-assisted extraction with protic ionic liquids was evaluated as a method to obtain phycobiliproteins. The optimal solvent was a 2-HEAA + 2-HEAF mixture at a pH of 7.0, with a power of 62 W and a biomass-to-solvent ratio of 10 mL/g. The extraction process lasted for 2 min and yielded concentrations and purities of 1.33 g/L, 0.84 g/L, and 0.41 g/L of allophycocyanin, phycocyanin, and phycoerythrin, respectively [164]. An emerging physical extraction technique, pulsed electrical field (PEF), is non-thermal, energy-efficient, and environmentally benign. Pulse discharge may induce electroporation or electro-disintegration of the cell membrane, thereby facilitating the breakdown of the cell homeostasis structure and the subsequent release of PBPs. In one study, phycocyanin was extracted from *A. maxima* using a combination of freezing and PEF treatment; the resulting extract had a purity of 2.45 and a concentration of 47 mg/g phycocyanin. The extraction process lasted for two hours and utilized an electrical field intensity of 25 kV/cm with a specific energy of 100 kJ/kg [165]. In addition to spark discharges, the utilization of high-voltage discharge-generated plasma to rupture the cell wall is an effective technique. The disruption of the cell wall caused by the spark discharge, which includes the production of reactive substances, strong shock waves, electrical fields, and ultraviolet radiation, facilitates the extraction of proteins. Sonication and spark discharge disintegration were utilized in one study to facilitate the extraction of phycocyanin from *Cyanidium caldarium* red algae. Continuous sonication demonstrated itself to be the most efficacious extraction technique in relation to the absolute yield of phycocyanin (53 mg/g). Despite a 4 mg/g decrease in yield when compared to ultrasound, a 30-min spark discharge extraction improved the integrity of the extracted proteins, as evidenced by a protein analysis that detected fewer modifications and generated greater purity than ultrasonication [166].

PBPs are typically extracted and subsequently purified using ammonium sulfate fractionation or ultrafiltration adsorption, depending on their charge ion properties, molecular weight, and solubility, after cell disruption, solid–liquid separation, and deslagging. Precipitation with ammonium sulfate is one of the most used techniques. Solid precipitates comprising different types of PBPs can be obtained by centrifuging the precipitates that arise when the concentration of ammonium sulfate in the solution is continuously elevated, thereby upsetting the stability of the PBP surface colloid. This causes the precipitation of PBPs in batches. A pilot process was conducted to produce, extract, and purify thermostable PC from *Synechocystis* sp. In optimizing the growth conditions, 75 mg/g of PC was obtained. Following chitosan-based flocculation, wet biomass was collected. Subsequent purification processes, including thermal treatment and ammonium sulfate precipitation in two phases, effectively eradicated chlorophyll and allophycocyanin contamination, producing a final purity of 2.9 [167].

Ultrafiltration uses a combination of membrane surface mechanical sieving, membrane pore blockage, and membrane adsorption to separate natural sensitive compounds, making it suitable for separating PBPs with a higher molecular weight. PBPs retain their general configuration, conformation, and optical activity due to the relatively mild nature of the treatment procedure. A purification method for phycobiliproteins containing certain biomolecules has been reported in *Nostoc commune*. The phycobiliprotein was extracted using the freeze-thaw technique, and the phycobiliprotein-containing solution was then individually permeated through two ultrafiltration membranes in crossflow mode. The results showed that the 150-kDa membrane’s cake layer was formed to filter the proteins in the solution containing phycobiliprotein, whereas the 10-kDa membrane’s pore blockage prevented the proteins from being filtered. Phycobiliprotein was successfully purified by the 10-kDa ultrafiltration membrane, as demonstrated by gel permeation chromatography. A 150 kDa UF membrane can determine the concentration of phycobiliprotein, while a 10 kDa membrane is capable of purifying the protein [168].

The efficiency of phycobilin extraction methods may vary depending on the marine cyanobacteria’s habitat and their adaptation to specific conditions. Cyanobacteria from extreme environments, such as hydrothermal vents or polar regions, may produce phycobilins with thermal or pH stability, expanding their industrial potential [169,170]. Future research should focus on characterizing PBs from non-conventional marine cyanobacteria, exploring their genetic and metabolic diversity to uncover new applications [20,171]. The conservation of these habitats and the implementation of sustainable cultivation practices are essential to ensuring the long-term availability of phycobilins for pharmaceutical, food, and cosmetic applications [24,170].

An appropriate extraction method must be selected based on the desired purity, yield, and intended application of the PBPs. Factors such as cell wall rigidity (in *Spirulina* or *Porphyridium*), extraction buffer composition, pH, and temperature significantly influence recovery rates [172]. Ultimately, combining mechanical pre-treatment with mild chemical or enzymatic protocols may yield optimal results while minimizing degradation.

A variety of methods have been developed for the extraction of PBPs, each with distinct advantages and limitations depending on the biological source, desired yield, and downstream applications. Table 6 summarizes the most commonly used PBPs extraction techniques, including mechanical, chemical, and green technologies, highlighting their benefits, drawbacks, and estimated recovery efficiencies.

### 6.2. Purification Approaches for Phycobiliproteins

Following extraction, PBPs require purification to remove pigments, lipids, and other cell components [173]. The most commonly used methods include ammonium sulfate precipitation, dialysis, and chromatographic techniques (e.g., ion exchange, gel filtration, and affinity chromatography). Purification aims to achieve high purity without compromising protein stability [173,174]. Chromatography offers excellent resolution and can selectively isolate specific PBPs, like phycocyanin (PC) or allophycocyanin (APC). However, it often involves higher operational costs and time. The choice of method depends on the intended application—e.g., analytical, pharmaceutical, or industrial—and the purity grade required [21,23].

The downstream phase of manufacture controls the quality of PBPs. Since PBPs are heat-sensitive materials, it is necessary to keep them away from high temperatures to avoid their breakdown. The equilibrium between production capacity and cost determines the degree of cell disruption and purification [4].

Maintaining the stability of PBPs during extraction is critical to preserve their biological activity and fluorescence properties [4]. Factors such as temperature, pH, ionic strength, and light exposure can influence protein integrity [23]. High temperatures during mechanical extraction can lead to denaturation, while extreme pH values may cause conformational changes or degradation [175].

Although new, highly efficient technologies that employ microwave, ultrasound, and pulsed electric fields are generally used in laboratories, they are not easily able to satisfy the needs of continuous, large-scale manufacturing [176]. On the other hand, pulsed spark discharge presents itself as a gentle and effective method of breaking down cell walls, which might find use in large-scale manufacturing [123].

Additionally, aqueous two-phase systems (ATPS) have demonstrated a potential for the mild and effective extraction of PBPs, maintaining their stability and bioactivity throughout the process [172]. As PBPs are water-soluble and often unstable under harsh purification conditions, ATPS and membrane-integrated processes have become increasingly favorable [177,178].

Future development aims to achieve industrial, continuous, automated manufacturing of PBs, where advances in membrane-based separation, eco-friendly flocculants, and mild cell disruption methods could bridge the gap between laboratory research and commercial production [158,179].

### 6.3. Safety Considerations: Presence of Cyanotoxins in Cyanobacterial Biomass

While phycobiliproteins derived from cyanobacteria offer significant biotechnological and pharmaceutical potential [180], it is critical to address the safety concerns associated with their biological source [181]. Certain cyanobacterial strains are known to produce secondary metabolites called cyanotoxins, which include microcystins, nodularins, cylindrospermopsin, anatoxins, and saxitoxins [181,182]. These toxins can have hepatotoxic, neurotoxic, dermatotoxic, or cytotoxic effects, posing serious health risks to humans, animals, and the environment [182,183].

The presence of cyanotoxins in cyanobacterial biomass intended for use in food, medicine, cosmetics, or veterinary applications necessitates rigorous quality control [184]. It is essential to screen the biomass for the presence of these harmful compounds before downstream processing [185]. The accumulation of cyanotoxins can occur under certain environmental stress conditions such as nutrient limitation, temperature fluctuations, and light intensity changes, making regular monitoring during large-scale cultivation a critical requirement [183,184].

Various analytical techniques, including ELISA, HPLC, LC-MS/MS, and PCR-based methods, are available for the detection and quantification of cyanotoxins [185,186]. Moreover, cultivation under controlled laboratory or photobioreactor conditions using non-toxic or well-characterized strains can significantly minimize this risk. Downstream purification processes such as activated carbon filtration or molecular separation techniques may also contribute to the removal of residual toxins [185,186].

Therefore, while PBPs hold considerable promise for therapeutic and industrial applications, addressing the safety implications of cyanotoxin contamination is crucial [182,186]. Ensuring the absence of these toxic metabolites will be a vital step in promoting the regulatory acceptance and safe commercialization of cyanobacteria-derived products [186].

## 7. Commercial Products from Phycobiliproteins and Economic Aspects

Currently, the biorefinery market, along with algae and microalgae-derived products, is experiencing significant growth. According to estimates by Future Marketing Insights, the commercial value reached USD 11.8 billion in 2023, with a compound annual growth rate (CAGR) of 8%. It is projected that by 2033, this market will reach a commercial value of USD $25.5 billion.

Regarding the quantity of biomass as the final product, microalgae-derived products can be classified into four main groups:

1. Microalgae used in bioremediation for treating wastewater, soil, and capturing CO_2_ from the atmosphere.
2. Production of biofuels and biofertilizers, using both whole biomass and extracts thereof.
3. Utilization as ingredients in human and animal nutrition.
4. Extraction of high-value products or compounds from microalgae, such as carotenoids, polyunsaturated fatty acids, and phycobiliproteins.

The latter have a wide range of applications and commercial value, from their use as food and cosmetics additives or colorants to their incorporation into fluorescence assays for research or diagnosis purposes [187].

The global commercial value of products containing phycobiliproteins reached USD 91.29 million in 2023. According to Market Research estimates this value is expected to increase to USD 290.81 million by 2029, with a CAGR of 21.3%, which is more than double the projected CAGR for the total microalgae market.

These markets include dietary supplements (Table 7) made from microalgae biomass that produce phycobiliproteins such as *Aphanizomenon flos-aquae*, *Arthrospira platensis*, *Spirulina* sp. (commonly marketed as *Spirulina*)., *Phormidium* sp., *Lyngbya* sp., *Synechocystis* sp., and *Synechococcus* sp. *Cyanotech* is one of the leading companies in this field.

The use of *Spirulina* has been documented for over 1000 years and has been established as a dietary supplement for over 40 years. Among its multiple active compounds is the blue protein C-PC. The use of extracts from *Spirulina* with high C-PC content were FDA-approved in 2013. This includes microalgae-based food colorants (Table 8), which have been used in various desserts such as glazes, ice creams, dessert toppings, powdered concentrates for beverages, dairy products, jellies, and cereals, among others. Microalgae are one of the primary natural photosynthetic pigment producers, especially chlorophylls, carotenoids, and phycobiliproteins. As shown in Table 9, PBPs have unique colors and molecular structures, and beneficial effects on human health [188].

## 8. Conclusions

Phycobiliproteins, with their diverse types, extraction processes, biological applications, activities, and potential health benefits, represent a burgeoning field of research. As understanding deepens and technologies advance, phycobiliproteins will likely play a crucial role in various scientific and industrial domains, contributing to sustainable and innovative solutions. The versatility and potential of phycobiliproteins across various applications, particularly in pharmaceuticals, underscores the need for continued research and technological advancements. Unleashing their full potential holds promise for innovative solutions, improved healthcare, and sustainable practices in diverse industries. Integrating phycobiliproteins into drug delivery systems and therapeutic strategies offers exciting prospects for addressing complex health challenges effectively and advancing the field of biomedicine.

## 9. Future Perspectives

PBs represent a significant role in the food, cosmetic, and biomedical industries as biological resources. Consequently, production of PBPs on a large scale is required, and the yield of PBs depends on the selection of high-yield algae strains. An appropriate reactor must be chosen that considers the conditions that must be used for the reactor to achieve high efficiency, taking into account the development habits and production scale requirements of the algae strains. Without a doubt, the most effective culture method for optimizing PBP accumulation is the mixotrophic strategy. Utilizing seawater and industrial effluent as sources of nutrients is an environmentally friendly way to accomplish cost-effective multifaceted resource utilization. However, algal development may be impacted by wastewater’s high salt and pollution levels. While employing chemical flocculation to collect biomass has shown significant benefits, physical machinery for algal biomass recovery is costly [189]. Cheap flocculants for industrial applications will be developed soon. When dewatering and drying, special attention should be paid to the balance between energy efficiency and consumption, as well as the potential loss of PBPs as a result of the harsh treatment conditions.

The downstream phase of manufacture controls the quality of PBPs. Since PBPs are heat-sensitive materials, it is necessary to keep them away from high temperatures to avoid their breakdown. The equilibrium between production capacity and cost determines the degree of cell disruption and purification. Although new technologies with great efficiency that employ microwave, ultrasound, and pulsed electric fields are generally used in laboratories, they are not easily able to satisfy the needs of continuous, large-scale manufacturing. On the other hand, pulsed spark discharge presents itself as a gentle and effective method of breaking down cell walls, which might find use in large-scale manufacturing. Hydrophobic chromatography, ion chromatography, and other techniques are commonly used for purification; however, they require time. Membrane chromatography offers stronger application prospects since it is faster and simpler to scale up. While PBP purity and yield are significant parameters, large-scale manufacturing also has to take economic rewards into account. Furthermore, a problem in lab-size research might have a bigger effect on industrial output. Before implementing a large-scale practice, pilot studies are required.

In addition, the development of synthetic biology approaches provides new opportunities to optimize PBs biosynthesis. Through genetic engineering, it is possible to construct recombinant strains with enhanced productivity, increased chromophore content, or tailored protein subunit composition. The use of metabolic engineering strategies in model organisms, such as *E. coli* or *Synechocystis*, is currently being explored to redirect metabolic fluxes towards phycobiliprotein pathways, offering an alternative to conventional algal cultivation. Moreover, CRISPR-Cas tools and genome-scale metabolic models can be applied to refine these strains further and accelerate strain improvement. From an industrial perspective, integrating these engineered strains into bioreactor systems optimized for cost-effective and scalable PBs production is essential to meet global demands. Addressing challenges related to scaling-up, downstream processing, regulatory acceptance, and production costs will be pivotal for the transition from bench to market. Ultimately, future development aims to achieve industrial, continuous, and automated manufacturing of PBs through a synergy of biotechnology, process engineering, and sustainability principles.

## Figures and Tables

**Table 1:** Mechanisms of action of phycobiliproteins (PBPs) and their interaction with various bioactivities and chronic diseases.

Activities	Action Mechanism of PBPs	Refererence
Antioxidant	Trapping ROS	[47,48]
Anticancer activity	Deregulates the expression of proinflammatory genes and components.	[49,50]
Multiple sclerosis	Has a protective effect on axonal structure loss	[51]
Diabetes	Decreases and/or suppresses NADPH oxidase activity	[52,53]
Atherosclerosis	Regulates atheroprotective activity, through molecular markers related to oxidative stress.	[54]

NADPH: Nicotinamide Adenine Dinucleotide Phosphate, ROS: Reactive Oxygen Species.

**Table 4:** Antitumor activity of PBPs.

Organism	Assay Results	Reference
Phycocyanin (PC)		
*S. platensis*	PBPs have shown dose-dependent antiproliferative activity against the MCF7 breast cancer cell line, with inhibition rates of 58, 60, and 64% at 400, 800, and 1600 μg/mL concentrations, respectively.	[70]
*Limnothrix* sp. NS01	The IC_50_ values obtained were 5.92, 5.66, and 4.52 μg/mL at 24, 48, and 72 h, respectively, against the MCF7 breast cancer cell line. Flow cytometry analysis (Annexin V/PI) determined that cell death occurred via apoptosis, confirmed by the reduced expression of Bcl-2, Stat 3, and Cyclin D1.	[131]
*S. platensis*	Dose-dependent activity was observed at concentrations of 2.5, 5, 7.5, and 10 μM against the NSCLC cell lines H358, H1650, and LETP-a2. At 7.5 μM, a G1 cell cycle arrest was induced, and migration was reduced from 69.72% to 44.65% in all three cell lines. Apoptosis was confirmed via flow cytometry (Annexin V/PI). The treatment downregulated the transcription levels of RIPK1/NFκB, exerting antiproliferative and antimigratory effects.	[49]
*S. platensis*	A dose-dependent activity was observed against the NSCLC cell lines H1975, H1650, and LTEP-a2, showing enhanced efficacy at 2, 4, 6, and 8 μM. A 59.21% cell cycle arrest was induced in the G1 phase compared to the control (42.23%). Early apoptosis was observed at 7.15%, while late apoptosis was 19.6% at 6 μM. C-PC reduced Bcl-2 expression, increased Bax expression, and downregulated the transcription of TIRAP/NFκB.	[132]
N/D	A concentration of 200 μg/mL of C-PC significantly improved the efficacy of radiotherapy in all cancer cell lines: CT-26 (mouse colon cancer), DLD-1 (human colon cancer), HT-29 (human colon cancer), and CRL-1831 (normal human colon cells). The treatment of normal colonic cells with C-PC did not show a significant impact (*p* > 0.05) on the radiation effects on these cells. Administration of C-PC at a concentration of 300 μg/mL reduced the expression of COX-2 mRNA by 58% and protein levels by 68%. (This enzyme is involved in radio resistance in tumor cells, causing inhibition of apoptosis, resistance, and proliferation.) The administration of 50 mg/kg to Balb/c mice for 30 days did not alter the body weight of the mice and showed no signs of toxicity or tissue damage (lungs, liver, kidneys, brain, and spleen) upon histopathological examination.	[133]
*Limnothrix* sp. KNUA002	C-PC showed no toxicity against HEI-OC1 cells at a concentration of 5 μg/mL. Treatment with PC at 1 and 2 μg/mL improved cell viability by 62% compared to 40% in control cells exposed to cisplatin. A 7% reduction in cell cycle arrest at the sub-G0/G1 phase was observed in cells pretreated with 1 μg/mL of PC. Additionally, treatment with PC at 30 μM induced the expression of the anti-apoptotic protein Bcl-2, while the expression of the pro-apoptotic protein Bax was lower in the phycocyanin treatment compared to cisplatin treatment.	[134]
N/D	C-PC treatment (10, 20, 40, and 80 μg/mL) protected H9c2 cells from intrinsic apoptosis induced by OGD/R (oxygen–glucose deprivation/reoxygenation) by modulating cytochrome c, apoptotic protease activating factor-1, and suppressing the phosphorylation of extracellular signal-regulated kinase and c-Jun N-terminal kinase. These results suggest that C-PC protects cardiomyocytes from ischemic damage by affecting mitochondrial fission and fusion dynamics, reducing apoptosis, and thus showing potential as a prophylactic or therapeutic agent for ischemic heart disease.	[135]
*Galdieria phlegrea* (009)	The IC_50_ values reported for various cell lines are as follows: against A431 epidermoid carcinoma cells, 9.8 ± 0.07 µM; against HaCat keratinocytes, >10 µM; against SV40-transformed mouse SVT2 cells, 3.7 ± 0.14 µM; and against Balb/c 3T3 mouse fibroblasts, >10 µM.	[136]
*S. platensis*	The IC_50_ value of 387.12 ± 0.34 μg/mL was observed against the Hep-G2 liver cancer cell line, with no activity detected against the Vero cell line.	[72]
*Limnothrix* sp. 37-2-1	The compound exhibits anticancer activity with a reduction of >65% at concentrations of 500 μg/mL. It promotes the upregulation of pro-apoptotic proteins such as BAX and Apaf-1, along with the activation of caspases 8, 9, and 3. Furthermore, a decrease in the expression of anti-apoptotic proteins such as Bcl-2, Mcl-1, and surviving was demonstrated.	[137]
*S. platensis*	Phycocyanin reduced lipid accumulation in L02 steatosis cells and in the liver of mice with non-alcoholic steatohepatitis (NASH), improving the antioxidant capacity of the liver. Western blot analysis showed an increase in AMPK phosphorylation expression and a reduction in the expression levels of SREBP-1c and its target genes, ACC and FAS. Additionally, there was an increase in the expression of the transcription factor PPAR-α, regulated by AMPK, and its target gene CPT1. Phycocyanin promoted AMPK phosphorylation in hepatocytes, while increasing the phosphorylation levels of ACC both in vivo and in vitro. Furthermore, it improved liver inflammatory infiltration by upregulating PPAR-γ and downregulating CD36, IL6, and TNF-α. These results suggest that phycocyanin can improve lipid accumulation and inflammation in mice with non-alcoholic fatty liver disease via the AMPK pathway in hepatocytes.	[138]
*Thermosynechococcus elongatus*	Cytotoxic activity against breast cancer (MCF7, IC_50_ of 158.9 μM), colorectal cancer (Caco-2, IC_50_ of 258.3 μM), and liver cancer (HepG2, IC_50_ of 277.5 μM).	[139]
*A. platensis* (biomass)	An IC_50_ >150 μg/mL was reported for cancer cell lines of glioblastoma (SF295), colorectal cancer (HCT116), prostate cancer (PC3), and an IC_50_ of 112.6 μg/mL for leukemia (HL60).	[96]
*S. platensis* MK343101	Inhibitory activity in HeLa cells of 64.1% at 500 μg/mL.	[99]
*S. platensis*	The expression of IRS-1 significantly decreased after treatment with 4.8 μM phycocyanin in lung cancer cells A549, H1299, and LTEP-a2, confirming transcriptome results via Western Blot. These findings suggest that IRS-1 may play a role in the antineoplastic function of phycocyanin in NSCLC cells.	[140]
N/D	Antitumor activity (50 mg/kg) in an in vivo 4T1 breast cancer model in Balb/c mice. Tumor growth was inhibited 12 days post-treatment, and by day 21, the tumor volume was 2.73 times smaller than the untreated control. Histopathological analysis showed inhibition of metastatic cancer cells in the lungs and liver compared to the control. Additionally, there was an increase in the survival rate of mice over 22 days.	[141]
*S. platensis*	Cytotoxic activity with an IC_50_ of 1.56 mg/mL in the MCF-7 breast cancer cell line. Treatment with 1.56 mg/mL induces upregulation of Bax and Cas-3 genes, along with downregulation of Bcl-2 expression.	[142]
*S. platensis*	Anticancer activity with IC_50_ values of 58.9, 48.1, and 44.7 µg/mL for the MCF-7, HCT-116, and HepG2 cell lines, respectively. C-PC induces apoptosis in the MCF7 cell line, with an increase in Cas9 protein (8.97 pg/mL) and a reduction in BCL2 (2.16 pg/mL) compared to the untreated control (Cas9 2.16 pg/mL and BCL2 5.10 pg/mL).	[102]
*A. platensis*	Commercial C-PC at 10 mg/mL reduced cell viability to <20% in fibroblasts and keratinocytes. Isolated C-PC reduced cell viability by >20% at 0.16 mg/mL for fibroblasts and at 0.62 mg/mL for keratinocytes. Isolated C-PC exhibited a pro-oxidant effect in keratinocytes when combined with UVA radiation exposure, increasing ROS levels, compared to commercial phycocyanin, which showed a protective effect with low ROS levels.	[104]
*S. platensis* SAM2021	It showed cytotoxic activity with an IC_50_ of 108 μg/mL against the colorectal cancer cell line HT-29.	[143]
C-Phycocyanin (C-PC), Allophycocyanin (APC)		
*S. platensis*	C-PC and purified APC can reverse TGFβ-induced migration of endometrial cancer cells and reduce peritoneal dissemination in a nude mouse model by modulating the TGFβ/SMAD4 signaling pathway. This involves the reduction of transcription factors such as TGFβR1, Smad4, Snail, SLUG, TWIST1/2, and ZEB1, followed by an increase in the expression of E-cadherin, while decreasing the expression of N-cadherin, vimentin, α-SMA, fibronectin, and TMEFF2 protein.	[144]
Phycoerythrin (PE)		
*G. corticata*	Cellular inhibition at 4.8 μg in SW-620 (42%) and HCT-116 (39%) cell lines.	[111]

PBPs: Phycobiliproteins, IC_50_: Half Maximal Inhibitory Concentration, MCF7: Michigan Cancer Foundation-7, Bcl-2: B-Cell Leukemia/Lymphoma 2, Cas-3: Caspase-3, Cas-9: Caspase-9, Stat 3: Signal Transducer and Activator of Transcription 3, NSCLC: Non-small cell lung cancer cells, RIPK1: Receptor-interacting Serine Threonine Protein kinase 1, NFκB: Nuclear Factor-Kappa B, BAX: Bcl-2-Associated X Protein, TIRAP: TIR Domain Containing Adaptor Protein, mRNA: Messenger RNA, COX-2: Cyclooxygenase-2, Apaf-1: Apoptosis protease-activating factor-1, Mcl-1: Myeloid Leukemia, AMPK: AMP-Activated Protein Kinase, SREBP-1c: Sterol regulatory element-binding protein 1, SREBP-1c: Sterol regulatory element-binding protein 1, ACC: Acetyl CoA Carboxylase, FAS: Fatty Acid Synthetase, PPAR-α: Peroxisome Proliferator Activated Receptor α, PPAR-γ: Peroxisome Proliferator Activated Receptor γ, CD36: Fatty Acid Translocase, IL6: Interleukin-6, TNF-α: Tumour Necrosis Factor alpha, CPT1: Carnitine Palmitoyltransferase-1, TGFβ: Transforming Growth Factor Beta, IRS-1: Insulin Receptor Substrate 1, UVA: Ultraviolet Radiation type A, ROS: Reactive Oxygen Species, N/D: Not Described, SMAD4: Specific Receptor-regulated Mothers Against Decapentaplegic Homolog 4, TGFβR1: Transforming Growth Factor Beta 1, Snail: Zinc Finger Protein SNAI1, SLUG: Zinc Finger Protein SNAI2, TWIST1/2: Twist-related Protein ½, ZEB1: Zinc Finger E-box Binding Homeobox 1, α-SMA: alpha smooth muscle actin, TMEFF2: transmembrane protein with an EGF-like and two Follistatin-like domains 2. OGD/R: Oxygen–Glucose Deprivation/Reoxygenation, NASH: Non-Alcoholic Steatohepatitis.

**Table 5:** Antibacterial and Antifungic Activity of PBPs.

Organism	Assay Results	Reference
Phycocyanin (PC)		
*Arthrospira* sp.	The gelatin-C-phycocyanin (C-PC) formulation exhibited antibacterial activity against *Staphylococcus aureus, Micrococcus luteus, E. coli*, and *Pseudomonas* sp., in a dose-dependent manner, with the best activity observed at 5 mg/mL C-PC, resulting in inhibition halos of 12.5, 18, 13, and 9 mm, respectively.	[92]
N/D	A MIC of 125 μg/mL was determined against *Enterococcus faecalis*.	[146]
*Oscillatoria minima*	Inhibition at 16 μg/mL against *Pseudomonas fragi*, *Pseudomonas vulgaris*, *Bacillus subtilis*, *Klebsiella oxytoca*, *Streptococcus pyogenes*, and against the algae *Nostoc*, *Gleocapsia*, and *Spirulina*.	[94]
*S. platensis* MK343101	It exhibited antibacterial activity with inhibition zones of 20 ± 0.9, 11 ± 0.7, 11 ± 0.5, and 11 ± 0.4 mm against *Shigella dysenteriae*, *Salmonella typhi*, *Pseudomonas aeruginosa*, and *Bacillus subtilis*, respectively. In comparison, tetracycline (30 μg) showed inhibition zones of 18 ± 0.3, 11 ± 0.5, 04 ± 0.5, and 11 ± 0.5 mm, respectively.	[99]
*A. platensis*	Antimicrobial activity against *Bacillus subtilis* ATCC 6633 (24.4% inhibition, 17.2% control), *Enterococcus faecalis* ATCC 29,212 (20.6% inhibition, 20.5% control), and *Streptococcus agalactiae* ATCC 13,813 (31.1% inhibition, 15.5% control).	[6]
Allophycocyanin (APC)		
*C. officinalis*	*S. aureus* ATCC 25,923 33.7% (33.6% control), *B. subtilis* ATCC 6633 32.6% (17.2% control), *E. faecalis* ATCC 29,212 31.5% (20.5% control), *S. agalactiae* ATCC 13,813 33.7% (15.5% control)	[6]
Phycoerythrin (PE)		
*Nostoc* sp. FA1	It showed activity against *B. subtilis* with an inhibition zone of 10.5 ± 0.28 mm and against *Candida albicans* with an inhibition zone of 10.98 ± 0.006 mm.	[147]
*Nostoc* sp. A5	Inhibition at 710 μg/mL with inhibition zones of 10.9 ± 0.16, 12.16 ± 0.44, 10.3 ± 0.88, 12.33 ± 0.33, 9.83 ± 0.44, and 12.5 ± 0.76 mm against *B. subtilis*, *Bacillus cereus*, *E. coli*, *S. aureus*, *Pseudomonas aeruginosa*, and *Salmonella typhimurium*, respectively. Antifungal activity (719 μg/mL) with inhibition zones of 13.55 μg/mL and 12.33 μg/mL against *Aspergillus niger* and *Candida albicans*.	[109]
*G. corticata*	Antifungal inhibition at 0.15 mg/mL against *C. albicans* (43 mm) and antimicrobial inhibition against *Clostridium perfringens* (35 mm), *S. aureus* (30 mm), *Shigella sonnei* (30 mm), *S. typhi* (30 mm), *P. aeruginosa* (29 mm), *E. coli* (27 mm), and *Bacillus cereus* (21 mm).	[111]

PBPs: Phycobiliproteins, C-PC: C-Phycocyanin, ATCC: American Type Culture Collection, N/D: Not Described, MIC: Minimum Inhibitory Concentration, mm: millimeter.

**Table 6:** Summary of commonly used extraction methods for phycobiliproteins (PBPs), and their advantages, disadvantages, and approximate yield.

Method	Principle	Advantages	Disadvantages	Estimated Yield/Notes	References
FTC	Disruption via ice crystal formation	Simple, no reagents required	Time-consuming, incomplete lysis	Moderate yield	[154,157]
Sonication	Ultrasound waves to disrupt cell membranes	Fast, efficient for small volumes	Heat generation, protein denaturation risk	High yield if controlled properly	[154,155]
Enzymatic digestion	Cell wall degradation using lysozyme or cellulase	Gentle, preserves bioactivity	Expensive, slow	Variable, strain-dependent	[156]
Osmotic shock	Cell bursting via hypotonic solutions	Mild, no chemicals	Inefficient for thick-walled cells	Low yield	[157]
UAE	Acoustic cavitation facilitates cell disruption	High efficiency, scalable, less solvent	Requires optimization, may generate heat	High yield (up to 80% PBPs recovery)	[155,158]
MAE	Rapid heating of intracellular water	Fast, low solvent usage	Risk of overheating or protein denaturation	High, but may affect purity	[158]
PEF	Electroporation of membranes	Low temperature, gentle on proteins	Requires specialized equipment	Moderate to high, depends on strain	[158,172]
Mechanical homogenization	High-shear mechanical disruption	Common in industry, scalable	Heat generation, high energy cost	Moderate to high	[154,172]
Chemical lysis (detergents, buffers)	Use of surfactants and buffers	Effective in lysing cells, customizable	May affect protein integrity	Variable, depends on composition	[156]

FTC: Freeze-thaw cycles, UAE: Ultrasound-assisted extraction, MAE: Microwave-assisted extraction, PEF: Pulsed electric field.

**Table 7:** Commercial food supplements available in the market that use phycobiliproteins, accessed in February 2025.

Product Name	Application	Country	Commercial Brand	Reference
Nutrex Hawaii Pure Hawaiian *Spirulina* Pacifica	*Spirulina* powder (Phycocyanin as main ingredient)	United States	Nutrex Hawaii Founded by Cyanotech	https://www.cyanotech.com/
*Spirulina* Gold Plus	*Spirulina* Tablets	United States	Earthrise Nutritionals	https://www.earthrise.com/
Organic *Spirulina* Powered *Truth in Food*	Phycocyanin Powder (as colorant or supplement)	Italy	Parry Nutraceuticals (sold by California Gold Nutrition^®^)	https://www.parrynutraceuticals.com/
E3 AFA blue green algae	Blue-Green Algae Supplements	United States	E3Live	https://www.e3live.com/
Klamin	Algae-based Food Supplements	Italy	NUTRIGEA Srl	https://nutrigea.com/
Numerous brands and products	Phycocyanin Extracts (as an ingredient in high-performance supplements)	United States	Cyanotech	https://www.cyanotech.com/
*Spirulina* algae powder	Phycobiliproteins naturally within the *Spirulina* algae.	United Kingdom	Organic Burst	https://www.organicburst.com/
*S. platensis*	Sunfood	United States	Sunfood	https://www.sunfood.com/
*Spirulina maxima*	Phycobiliproteins naturally within the *Spirulina* algae.	China	Hainan Super Biotech Co., Ltd.	https://www.super-biotech.com/
Blue *Spirulina* E10, 18, and 25	*Spirulina* extract (Phycocyanin as main ingredient)	China	Zhejiang Binmei Biotechnology Co., Ltd.	https://www.binmei-global.com/

Co.: Company, Ltd.: Limited, Srl: società a responsabilità limitata.

**Table 8:** Phycocyanin commercial food colorants available in the market, accessed in February 2025.

Product Name	Application	Country	Commercial Brand	Reference
Blue *Spirulina* Extract	Phycobiliproteins are extracted from *Spirulina* algae, providing a natural blue colorant.	United States	E3Live	https://www.e3live.com
Blue Majik Powder	Derived from Phycocyanin, a type of Phycobiliprotein found in *Spirulina*, providing a vibrant blue color.	United States	E3Live	https://www.e3live.com
Phycocyanin Extract	Extracted from *Spirulina* algae, Phycocyanin serves as a natural blue food colorant	China	Tianjin Norland Biotech Co., Ltd.	https://www.norlandbiotech.com
Blue *Spirulina* Extract Powder	Contains phycocyanin, a Phycobiliprotein sourced from *Spirulina*, providing a vivid blue hue to food products.	Taiwan	FEBICO	https://www.febico.com
Blue-Green Algae Powder	Phycobiliproteins extracted from *Spirulina* algae contribute to the blue-green color of this natural food coloring agent.	United States	Gourmet Imports	https://www.gourmetimports.com
Phycocyanin Blue Powder	Derived from phycocyanin, a Phycobiliprotein from *Spirulina*, this powder serves as a natural blue food colorant	China	Qingdao BNP BioScience Co., Ltd.	https://www.bnpharma.com
Blue Algae Powder	Phycobiliproteins from *Spirulina* algae contribute to the blue color of this natural food coloring product.	Australia	Southern Biological	https://www.southernbiological.com
Organic Blue Spirulina Powder	Contains phycocyanin, sourced from *Spirulina*, providing a natural blue hue to food and beverages	Mexico	NineLife	https://ninelife.mx
Blue Spirulina Blue Powder	Organic phycocyanin from *Arthrospira platensis*serve as a natural blue food colorant in this powder form.	China	Shaanxi honghao Bio-tech Co.,ltd,	https://www.herb-extract-supplier.com
Blue Phycocyanin Extract	Phycocyanin, derived from *Spirulina*, is used as a natural blue food colorant in various food applications.	Israel	Algatechnologies Ltd.	https://www.algatech.com
LinaBlue^®^	Pigment of phycocyanin derived from *Spirulina*, used as a natural food colorant as well as a functional food ingredient.	United States	Sun Chemical ©	https://www.sunchemical.com/linablue/
Organic Blue *Spirulina*	*Spirulina* powder extract (Phycocyanin as main ingredient)	China	Zhejiang Binmei Biotechnology Co., Ltd.	https://www.binmei-global.com/

Co.: Company, Ltd.: Limited.

**Table 9:** Commercial phycobiliproteins fluorescent markers and diagnostic reagents available in the market, accessed in February 2025.

Product Name	Application	Country	Commercial Brand	Reference
Allophycocyanin-Conjugated Antibodies	Provides a variety of fluorescently labeled antibodies and reagents, including those incorporating phycobiliproteins, for flow cytometry and other research applications.	United States	BD Biosciences	https://www.bdbiosciences.com
Phycoerythrin-Conjugated Antibodies	Antibodies and proteins for research use, conjugated to phycobiliproteins for detection purposes. Immunofluorescence assays	United States	Santa Cruz Biotechnology, Inc.	https://www.scbt.com
Phycoerythrin-Conjugated Antibodies	Antibodies and protein conjugates for various research applications, Immunofluorescence assays	United States	Rockland Immunochemicals Inc.	https://www.rockland-inc.com
Phycoerythrin-Conjugated Antibodies	Antibodies and proteins, some of which may be conjugated with phycobiliproteins for fluorescence-based assays, Immunofluorescence assays	United States	ProSci Inc.	https://www.prosci-inc.com
Phycoerythrin-Conjugated Antibodies	Research antibodies and reagents, which may include some labeled with phycobiliproteins for use in fluorescence-based experiments.	United States	Cell Signaling Technology, Inc.	https://www.cellsignal.com
Phycoerythrin-Conjugated Antibodies	Wide range of research reagents, including antibodies and proteins, conjugated with phycobiliproteins for fluorescent labeling.	United States	Sigma-Aldrich Corporation (Merck).	https://www.sigmaaldrich.com
Phycoerythrin-Conjugated Antibodies	Extensive catalog of antibodies and research tools, including some labeled with phycobiliproteins for various applications.	United Kingdom	Abcam plc.	https://www.abcam.com
Cy7-Phycoerythrin Conjugate R-phycoerythrin	Diagnostic application: Immunofluorescence assay	Germany	Chroma Gesellschaft Schmidt & Co. GmbH.	https://www.chroma.de
Phycoerythrin-Conjugated Antibodies	Flow cytometry and immunofluorescence assays	United States	BioLegend	https://www.biolegend.com
Phycoerythrin-Antibody Conjugates	Flow cytometry and immunofluorescence assays	United States	Thermo Fisher Scientific.	https://www.thermofisher.com

BD: Becton Dickinson, Co.: Company, Inc.: Incorporated, GmbH: Gesellschaft mit beschränkter Haftung, plc: Programmable Logic Controller.

## Data Availability

Not applicable.

## Abbreviations

The following abbreviations are used in this manuscript:

APC	Allophycocyanin
PBP	Phycobiliprotein
PBs	Phycobilins
PBSs	Phycobilisomes
PC	Phycocyanin
PE	Phycoerythrin
PEC	Phycoerythrocyanin
PEF	Pulsed electrical field
IC_50_	Half Maximal Inhibitory Concentration
SC_50_	Half Scavenging Concentration
g/mL	Grams per milliliter
N/D	Not described.
mm	Millimeter
mg/mL	Milligram per milliliter
UAE	Ultrasonic Assisted Extraction
μg/mL	Microgram per milliliter
SD	Standard deviation
